# Early Intestinal Ultrasound Predicts Clinical and Endoscopic Treatment Response and Demonstrates Drug-Specific Kinetics in Moderate-to-Severe Ulcerative Colitis

**DOI:** 10.1093/ibd/izad274

**Published:** 2023-11-27

**Authors:** Floris A de Voogd, Steven J Bots, Elsa A van Wassenaer, Maria de Jong, Maarten J Pruijt, Geert R D’Haens, Krisztina B Gecse

**Affiliations:** Department of Gastroenterology and Hepatology, Amsterdam University Medical Center, Location AMC, Amsterdam, the Netherlands; Department of Gastroenterology and Hepatology, Amsterdam University Medical Center, Location AMC, Amsterdam, the Netherlands; Paediatric Gastroenterology, Emma Children’s Hospital, Amsterdam UMC, University of Amsterdam, Amsterdam, the Netherlands; Department of Gastroenterology and Hepatology, Amsterdam University Medical Center, Location AMC, Amsterdam, the Netherlands; Department of Gastroenterology and Hepatology, Amsterdam University Medical Center, Location AMC, Amsterdam, the Netherlands; Department of Gastroenterology and Hepatology, Amsterdam University Medical Center, Location AMC, Amsterdam, the Netherlands; Department of Gastroenterology and Hepatology, Amsterdam University Medical Center, Location AMC, Amsterdam, the Netherlands

**Keywords:** intestinal ultrasound, ulcerative colitis, treatment response

## Abstract

**Background:**

Intestinal ultrasound (IUS) is an emerging modality in monitoring disease activity in ulcerative colitis (UC). Here, we aimed to identify early IUS predictors of treatment response as evaluated by endoscopy and assessed the kinetics of IUS changes.

**Methods:**

This prospective, longitudinal study included UC patients with endoscopic disease activity (endoscopic Mayo score [EMS] ≥2) starting anti-inflammatory treatment. Clinical scores, biochemical parameters and IUS were assessed at baseline (W0), at week 2 (W2), at W6(W6), and at the time of second endoscopy (W8-W26). Per colonic segment, endoscopic remission (EMS = 0), improvement (EMS ≤1), response (decrease in EMS ≥1), and clinical remission (Lichtiger score ≤3) were assessed and correlated with common IUS parameters. Additionally, drug-specific responsiveness of bowel wall thickness (BWT) was assessed.

**Results:**

A total of 51 patients were included and followed, and 33 patients underwent second endoscopy. BWT was lower from W6 onward for patients reaching endoscopic improvement (3.0 ± 1.2 mm vs 4.1 ± 1.3 mm; *P* = .026), remission (2.5 ± 1.2 mm vs 4.1 ± 1.1 mm; *P* = .002), and clinical remission (3.01 ± 1.34 mm vs 3.85 ± 1.20 mm; *P* = .035). Decrease in BWT was more pronounced in endoscopic responders (−40 ± 25% vs −4 ± 28%; *P* = .001) at W8 to W26. At W6, BWT ≤3.0 mm (odds ratio [OR], 25.13; 95% confidence interval, 2.01-3.14; *P* = .012) and color Doppler signal (OR, 0.35; 95% confidence interval, 0.14-0.88; *P* = .026) predicted endoscopic remission and improvement, respectively. Submucosal layer thickness at W6 predicted endoscopic remission (OR, 0.09; *P* = .018) and improvement (OR, 0.14; *P* = .02). Furthermore, BWT decreased significantly at W2 for infliximab and tofacitinib and at W6 for vedolizumab.

**Conclusions:**

BWT and color Doppler signal predicted endoscopic targets already after 6 weeks of treatment and response was drug specific. IUS allows close monitoring of treatment in UC and is a surrogate marker of endoscopy.

**Key Messages**

**What is already known?**

Intestinal ultrasound is an emerging, noninvasive tool to assess treatment response in ulcerative colitis. Studies demonstrating its role early on in treatment follow-up are scarce.

**What is new here?**

Intestinal ultrasound is accurate to predict endoscopic treatment response, already after 2 to 6 weeks into treatment. Response to treatment is drug specific and intestinal ultrasound demonstrated specific response kinetics.

**How can this study help patient care?**

Intestinal ultrasound is accurate to determine disease activity, severity, and treatment response in ulcerative colitis. Furthermore, it predicts response early into treatment and is an attractive noninvasive alternative to endoscopy.

## Introduction

Ulcerative colitis (UC) is a chronic inflammatory disease of the bowel with a relapsing-remitting pattern. Endoscopic healing is associated with improved long-term outcomes^1^ and is therefore currently recommended as a treatment target.^2,3^ However, endoscopy is unattractive for frequent monitoring as it requires bowel preparation, is invasive, costly, and time-consuming.^2,4,5^ Noninvasive biomarkers such as C-reactive protein and fecal calprotectin (FCP) are widely used to objectively identify flares and to assess treatment response. However, they lack information on disease extent, and optimal cutoff values still vary.^6-8^ Recently, intestinal ultrasound (IUS) has become a noninvasive, low-cost and easily accessible alternative.^2,9-11^ IUS, most importantly bowel wall thickness (BWT), shows high accuracy when compared with endoscopy in diagnosing UC extending beyond the rectum, is able to differentiate among quiescent, mild, and moderate-severe disease^9,10,12^ and identifies disease extent.

Previous data showed that IUS detects treatment response in UC as soon as 2 weeks after treatment initiation, when compared with clinical outcomes.^11^ However, no studies have previously investigated the predictive role of early IUS for endoscopic outcomes.^13^ In this longitudinal cohort of patients with moderate-to-severe UC treated with drugs of different mechanism of action, we aimed to identify early IUS predictors of treatment response as evaluated by endoscopy. Furthermore, we assess the kinetics of IUS changes and correlate IUS findings with clinical, biochemical, and endoscopic outcomes.

## Methods

### Study Design

In this single-center prospective longitudinal study, patients ≥18 years of age with previously diagnosed UC were eligible for inclusion.^2^ Consecutive patients with moderate-to-severe UC according to the endoscopic Mayo score (EMS) (≥2) starting on anti-inflammatory treatment were included if IUS also confirmed the presence of disease activity in at least 1 segment. IUS disease activity was defined as a BWT >3.0 mm^9,10,13,14^ and 1 additional pathological parameter (color Doppler signal [CDS], loss of haustrations, loss of wall layer stratification, fatty wrapping, or mesenteric lymph nodes) or a BWT ≥5.0 mm in the rectum (Table 1). Subsequently, individual wall layers were measured at the area of highest BWT in the sigmoid colon. When wall layer stratification was extensively lost (score = 3) (Table 1) wall layers were measured at the nearest region in the sigmoid colon with BWT >3.0 mm and perseverance of wall layer stratification. Patients were excluded when there was no endoscopy and/or IUS performed at the start of treatment, if the window between baseline endoscopy and baseline IUS exceeded 4 weeks or when treatment was changed between baseline endoscopy and baseline IUS. Imminent need of surgery, presence of an infectious gastroenteritis, presence of cytomegalovirus infection, pregnancy, and obesity (body mass index >35 kg/m^2^) were also exclusion criteria. This study (NL7903, the Netherlands Trial Register) was approved by the medical ethical committee of the Amsterdam University Medical Center, all patients gave informed consent and quality of the data was assured by an independent monitor.

**Table 1.** Intestinal ultrasound parameters, outcomes, and definitions.

Parameter	Measurement	Pathologic definition
Bowel wall thickness	4 measurements from lumen-mucosa interface up to muscularis propria-serosa interface (2× measurement longitudinal (≥10 mm between measurements) + 2× measurement cross-sectional plane (≥90° between measurements))/4	≥3.0 mm for colonic segments ≥5.0 mm for rectum
Color Doppler signal	Categories 0: no Doppler signal 1: 1 or 2 single vessels 2: >2 single vessels or stretches limited to the wall 3: stretches extending into the mesentery	≥2, NA to rectum
Haustrations	Categories 1: preserved haustrations 2: loss of haustrations	2, NA to rectum
Wall layer stratification	Categories 1: preserved wall layer stratification 2: loss of wall layer stratification ≤30 mm in length 3: extensive loss of wall layer stratification >30 mm in length	≥2, NA to rectum
Fatty wrapping	Categories 1: absence of fatty wrapping 2: presence of fatty wrapping	2, NA to rectum
Mesenteric lymph nodes	Categories 1: absence of lymph nodes 2: presence of lymph nodes ≤5.0 mm in the shortest axis 3: presence of lymph nodes >5.0 mm in the shortest axis	3, NA to rectum
Disease activity per segment	NA	BWT >3.0 mm and ≥1 other pathological IUS parameter

Abbreviations: BWT, bowel wall thickness; IUS, intestinal ultrasound; NA, not applicable.

### Procedures

Patient demographics and medical history were collected at the time of the baseline IUS. At baseline, week 2 (W2), at W6, and the time of a second endoscopy between W8 and W26, the Simple Clinical Colitis Activity Index (SCCAI),^15^ Lichtiger score,^16^ C-reactive protein, hemoglobin, leukocyte count, thrombocyte count, serum albumin, and FCP were measured.

IUS examinations of all colonic segments were performed at baseline, W2, W6, and W8 to W26, all with an Epiq 5G ultrasound scanner (Philips) with C5-1 and L12-5 probes. Gain, frequency, and focus settings were optimized per patient for the best images. A velocity scale of 5 cm/s was used to measure CDS. All colonic segments were visualized starting at the sigmoid and moving proximally to the ascending colon. The rectum was visualized with the C5-1 probe and identified when we determined an anterior and posterior bowel wall with an air-filled lumen posterior to the prostate or uterus/vagina (Supplementary Figure 1). Cine loops including CDS, mesenteric fat, and lymph nodes were recorded for every colonic segment in longitudinal and cross-sectional plane. At the time of the IUS, the ultrasonographer (F.A.d.V). was blinded to biochemical and endoscopic disease activity and did not perform any of the endoscopies. All anonymized cine loops were stored per patient per time point and read more than a month after the last visit of the last patient. A second reader (K.B.G.) who was blinded for clinical, biochemical, and endoscopic data, read 30 randomly selected ultrasonographic examinations and scored all IUS parameters (Table 1) for the sigmoid colon. All data were entered in an electronic data management system.

A complete colonoscopy or sigmoidoscopy, as per discretion of the treating physician, was performed by an endoscopist at baseline and W8 to W26 and the disease extent was determined when possible. The window between IUS and endoscopy could not exceed 4 weeks, and treatment was not changed between endoscopy and IUS.

### Definitions and Outcomes

Clinical remission was defined as SCCAI ≤2^17^ or Lichtiger score ≤3.^17^ Clinical response was defined as a decrease SCCAI ≥3^17^ or decrease Lichtiger score ≥3.^17^

All IUS parameters were scored for each segment using a DICOM-viewer (RadiAnt DICOM Viewer, version 2016). IUS disease activity was established per segment when BWT >3.0 mm and 1 other pathologic parameter (Table 1) was present. Response per parameter was defined when it improved from pathologic to nonpathologic.

EMS was scored for each assessed segment separately. Endoscopic remission was defined as EMS = 0, endoscopic improvement as EMS ≤1, and endoscopic response as a decrease of EMS ≥1. Complete endoscopic remission and improvement was defined as EMS = 0 or ≤1 for all segments, respectively.

The primary outcome of the study was to investigate if BWT as measured by IUS is a surrogate marker of endoscopic improvement after 6 weeks of treatment.

### Secondary Outcomes

Secondary outcomes were differences between (Δ)IUS parameters at W2, W6, and W8 to W26 in sigmoid, descending, transverse, and ascending colon for patients reaching several clinical and endoscopic targets vs patients not reaching these targets. Furthermore, correlation between (Δ)IUS parameters with (Δ)EMS per segment was investigated. In addition, prediction and association for IUS parameters in combination with clinical and biochemical markers was tested for (segmental) endoscopic outcomes. Response of IUS parameters was also compared for different therapeutic agents.

### Sample Size Calculation

We performed a sample size calculation to detect a significant difference in BWT after 6 weeks of treatment for patients reaching endoscopic improvement vs no endoscopic improvement in the sigmoid colon. There are no studies available investigating early BWT changes according to endoscopic outcomes. A recent study found a difference of BWT 1.16  ± 1.60 mm in the sigmoid colon between clinical responders and nonresponders after 12 weeks of treatment.^11^ With a level of significance of α = 0.05 and 80% power, we needed 11 patients in at least 1 group to detect this difference. As we assumed 30% to 60% of patients to show no endoscopic improvement,^18-20^ we aimed for 33 patients receiving an endoscopy at W0 and W8 to W26 and continued inclusion when 33 patients had an endoscopy at W8 to W26.

### Statistics

SPSS Statistics for Windows, version 26 (IBM Corporation) was used for statistical analysis. Mean ± SD and median with interquartile range (IQR) were used to report normally and nonnormally distributed data. A (paired) *t* test, Mann-Whitney *U* test, Wilcoxon rank test, chi-square test, or McNemar test were used as appropriate. Area under the receiver-operating characteristic curve (AUROC) was used to determine accuracy, sensitivity, specificity, positive predictive value (PPV), and negative predictive value (NPV). Odds ratios (ORs) were calculated with logistic regression analysis, both for univariable and multivariable analysis (backward selection). Spearman’s correlation coefficient was used to determine correlation.^21^ Intraclass correlation coefficient and Cohen’s kappa (weighted when ≥3 categories) statistics were used to determine interobserver agreement.^22,23^ A *P* value of .05 was used to determine significance.

## Results

A total of 65 consecutive patients were screened, of whom 51 patients met all inclusion criteria (Table 2). At baseline endoscopy the proximal extent of active disease was reached in 33 (65%) patients. In the subgroup of patients in which proximal disease extent was not reached on endoscopy, IUS identified additional affected segments in 10 (20%) patients. A total of 33 (65%) patients underwent a second endoscopy between W8 to W26 (mean 18 ± 8 weeks  from baseline) with 1 ± 0.9 week between IUS and endoscopy (Supplementary Figure 2). A total of 18 (35%) patients were not scheduled for a second endoscopy, predominantly due to the reduced capacity as a consequence of the COVID-19 pandemic. At W8 to W26, 27 (53%) and 22 (43%) patients were in clinical remission according to the SCCAI and the Lichtiger score, respectively. Clinical response was seen in 33 (65%) and 36 (71%) patients, respectively. Of the patients undergoing a second endoscopy, 11 (33%) patients reached complete endoscopic improvement, with 4 (12%) patients reaching complete endoscopic remission. BWT showed moderate to good correlation with EMS for all colonic segments (except the rectum), at both baseline and W8 to W26 (Supplementary Figure 3). Subsequent analysis per colonic segment for endoscopic targets was primarily performed for the left-sided colon (sigmoid colon: n = 33; descending colon: n = 31) as most endoscopic data were available for these segments.

**Table 2.** Baseline characteristics (N = 51).

Female	28 (55)
Age at inclusion, y	38 (18-72)
**Montreal classification**	
Left-sided colitis (E2)	25 (49)
Pancolitis (E3)	26 (51)
**Medication history**	
Corticosteroids	47 (92)
Aminosalicylates	51 (100)
Thiopurines	32 (63)
Methotrexate	5 (10)
Previous biological and/or JAK inhibitor exposure	
0	24 (47)
1	12 (24)
2	15 (29)
**Newly introduced treatment at baseline**	
Systemic corticosteroids	40 (78)
Aminosalicylates	
Monotherapy	2 (4)
Combination therapy	29 (57)
Thiopurines	
Monotherapy	3 (6)
Anti-TNF combination therapy	15 (29)
**Biologics**	
Infliximab	18 (35)
Adalimumab	1 (2)
Vedolizumab	14 (27)
Ustekinumab	2 (4)
Tofacitinib	14 (27)
Ciclosporin	1 (2)
**Clinical and biochemical parameters**	
Simple clinical colitis activity index	9 (6-12)
Lichtiger score	12 (9-14)
CRP, mg/L	26.64 ± 42.45
Hemoglobin, mmol/L	7.63 ± 1.45
Leukocytes, 10^9^/L	9.43 ± 4.13
Thrombocytes, 10^9^/L	384 ± 140
Albumin, g/L	38.0 ± 6.5
Fecal calprotectin, mg/kg	2555 ± 2125
**Most proximal extent at baseline IUS**	
Sigmoid colon	8 (16)
Descending colon	16 (31)
Transverse colon	15 (29)
Ascending colon	12 (24)
**Most proximal disease extent at baseline endoscopy**	
Sigmoid colon	7 (14)
Descending colon	8 (16)
Transverse colon	4 (8)
Ascending colon	14 (27)
Proximal disease extent not reached	18 (35)
**IUS parameters sigmoid**	44 (86)
Bowel wall thickness, mm	4.98 ± 1.13
CDS (stretches within/extending bowel wall)	44 (86)
Loss of colonic haustrations	44 (86)
Loss of wall layer stratification	15 (29)
Presence of fatty wrapping	29 (57)
**Endoscopic Mayo score sigmoid**	
1	1 (2)
2	19 (70)
3	31 (61)

Values are n (%), median (interquartile range), or mean ± SD. Four patients treated with tofacitinib also participated in another study.^24^

Abbreviations: BWT, bowel wall thickness; CDS, color Doppler signal; CRP, C-reactive protein; IUS, intestinal ultrasound; TNF, tumor necrosis factor.

### Responsiveness of BWT per Endoscopic Target

**Figure 1.**
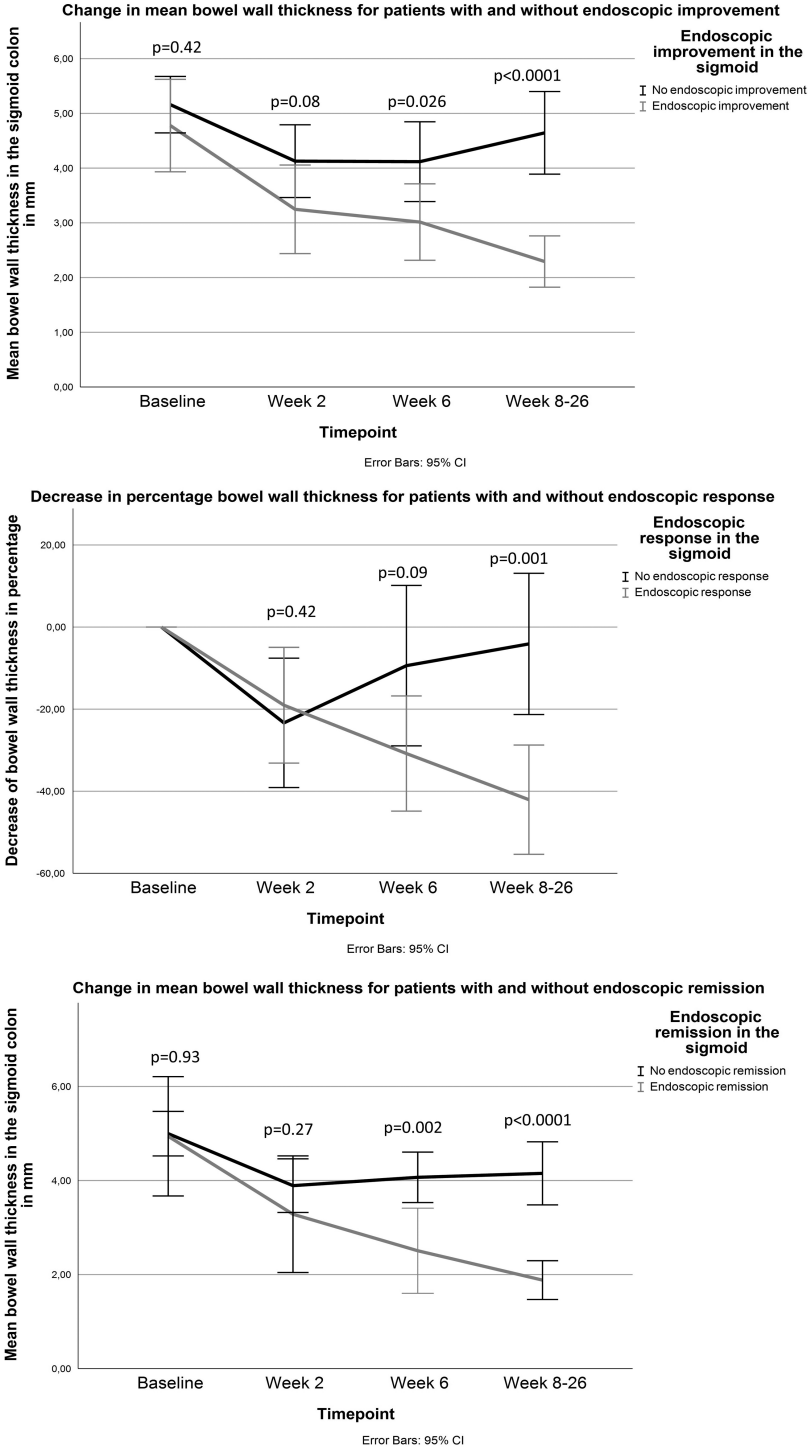
Bowel wall thickness per time point for patients with and without endoscopic improvement, endoscopic response, and endoscopic remission. Data are reported in Supplementary Table 1. CI, confidence interval.

The correlation between change of BWT and change of EMS was moderate for the sigmoid (ρ = 0.63, *P* < .0001) as well as for the descending colon (ρ = 0.54, *P* = .003). At follow-up, a total of 19 (58%), 16 (48%), and 10 (30%) patients had endoscopic response, improvement, and remission, respectively, in the sigmoid colon. From W6 onward, BWT was significantly different between patients with and without endoscopic improvement (3.0 ± 1.2 mm vs 4.1 ± 1.3 mm; *P* = .026) and endoscopic remission (2.5 ± 1.2 mm vs 4.1 ± 1.1 mm; *P* = .002) (Figure 1, Supplementary Table 1). Decrease in BWT was significantly different between patients with and without endoscopic response at W8 to W26 compared with baseline (−40% ± 25 vs −4% ± 28, *P* = .001) but not at W6 (Figure 1, Supplementary Table 1). Decrease in BWT was not significantly different between patients receiving endoscopy between W8 and W17 (n = 17) compared with endoscopy between W18 and W26 (n = 16; −22 ± 31% vs −30 ± 34%; *P* = .52).

### Optimal Early BWT Cutoff to Predict Endoscopic Targets

For the sigmoid colon, BWT ≤3.0 mm at W6 accurately predicted endoscopic remission in the sigmoid colon upon follow-up with 78% (95% CI, 40%-96%) sensitivity and 90% (95% CI, 65%-98%) specificity (AUROC, 0.82; 95% CI, 0.63-1.00; *P* = .007; PPV: 78%, NPV: 89%) (Supplementary Table 2, Supplementary Figure 4). For the descending colon, a BWT ≤3.6 mm (W2) and ≤3.2 mm (W6) accurately predicted endoscopic remission upon follow-up (Supplementary Table 3). Cutoff values to predict endoscopic improvement or response were not significant at W2 or W6 (Supplementary Tables 2 and 3).

### Optimal Bowel Thickness Cutoff at W8 to W26 to Determine Endoscopic Targets

A BWT of 2.7 mm demonstrated endoscopic remission in the sigmoid colon with 100% (95% CI, 60%-100%) sensitivity and 86% (95% CI, 64%-96%) specificity (AUROC, 0.95; *P* < .0001; PPV: 73%, NPV: 100%). For endoscopic improvement, 3.5 mm was most accurate with 92% (95% CI, 64%-100%) sensitivity and 87% (95% CI, 60%-98%) specificity (AUROC, 0.96; *P* < .0001; PPV: 87%, NPV: 93%). For endoscopic response, a decrease of 23% (AUROC, 0.81; 95% CI, 0.61-1.00; *P* = .019) in BWT was accurate with 77% (95% CI, 46%-94%) sensitivity and 81% (95% CI, 0.54%-0.95%) specificity (AUROC, 0.81; *P* = .019; PPV: 77%, NPV: 81%).

### FCP to Predict Endoscopic Targets

A FCP concentration ≤530 µg/g at W6 was accurate to predict endoscopic improvement in the sigmoid colon, with 78% sensitivity and 91% specificity (AUROC, 0.78; 95% CI, 0.52-1.00; *P* = .03). Endoscopic remission and response could not be accurately predicted with FCP at W6 (Supplementary Table 4). At W8 to W26, 183 µg/g (AUROC, 0.88; 95% CI, 0.72-1.00; *P* = .008) and 253 µg/g (AUROC, 0.88; 95% CI, 0.71-1.00; *P* = .003) were accurate to determine endoscopic remission and improvement in the sigmoid colon, respectively (Supplementary Table 4).

### Logistic Regression for IUS Parameters for Endoscopic Targets

In multivariate analysis, a BWT of 3.0 mm (OR, 25.13; 95% CI, 2.01-314; *P* = .012) or BWT per mm increase (OR, 0.14; 95% CI, 0.03-0.73; *P* = .02) predicted endoscopic remission at W6 in the sigmoid. Categorical CDS increase (OR, 0.35; 95% CI, 0.14-0.88; *P* = .026) predicted endoscopic improvement at W6 in multivariate analysis. At W8 to W26, BWT ≤3.5 mm (OR, 74.00; 95% CI, 4.72-1159; *P* = .002) or BWT per mm increase (OR, 0.05; 95% CI, 0.005-0.52; *P =* .012) detected endoscopic remission and improvement in the sigmoid colon, respectively (Table 3). At W6, return of colonic haustrations (OR, 13.50; 95% CI, 2.01-90.69; *P* = .007) was the only parameter that predicted endoscopic response in the sigmoid colon. At W8 to W26, 23% decrease in BWT (OR, 19.14; 95% CI, 1.68-218; *P* = .017]) or decrease per percentage BWT (OR, 1.05; 95% CI, 1.01-1.10; *P* = .034) in combination with change from presence to absence of mesenteric fat (OR, 12.56; 95% CI, 1.05-150.43; *P* = .046) detected endoscopic response. FCP cutoff values at W6 and W8 to W26 predicted and determined endoscopic improvement or remission but were not significant anymore in multivariate analysis (Table 3). For the descending colon, data are reported in Supplementary Tables 3, 5, and 6.

**Table 3.** Univariable and multivariable logistic regression analysis to predict endoscopic remission or endoscopic improvement at W6 and W8 to W26 in the sigmoid colon.

	Univariable		Multivariable	
	OR (95% CI)	*P* value	OR (95% CI)	*P* value
**Sigmoid W6**				
Bowel wall thickness (per mm increase)	0.30 (0.11-0.76)	**.011** ^**a**^	0.14 (0.03-0.73)	**.02** ^**a**^
Bowel wall thickness cutoff ≤3.0 mm	29.75 (3.47-255.00)	**.002** ^**a**^	25.13 (2.01-314)	**.012** ^**a**^
Color Doppler signal (per 1 category increase)	0.40 (0.15-1.01)	.053	0.75 (0.06-9.10)	.82
Loss of stratification	1.52 (0.21-11.23)	.68	—^b^	1.00
Loss of haustration	0.26 (0.04-1.57)	.14	8.38 (0.28-253.00)	.22
Presence of fatty wrapping	^a^	1.00	—^b^	1.00
Presence of lymph nodes	—^b^	1.00	—^b^	1.00
Submucosal wall thickness (per mm increase)	0.09 (0.01-0.65)	**.018** ^**a**^	0.45 (0.04-4.90)	.52
**Sigmoid W8-W26**				
Bowel wall thickness (per mm increase)	0.06 (0.007-0.58)	**.014** ^**a**^	0.05 (0.005-0.52)	**.012** ^**a**^
Bowel wall thickness cutoff ≤2.7 mm	—^b^	1.00	—^b^	1.00
Color Doppler signal (per 1 category increase)	0.53 (0.22-1.27)	.16	1.12 (0.18-7.09)	.90
Loss of stratification	0.91 (0.08-10.21)	.94	—^b^	1.00
Loss of haustration	—^b^	1.00	—^b^	1.00
Presence of fatty wrapping	—^b^	1.00	—^b^	1.00
Presence of lymph nodes	—^b^	1.00	—^b^	1.00
Submucosal wall thickness (per mm increase)	0.03 (0.002-0.52)	**.016** ^**a**^	0.38 (0.008-18.50)	.63
Fecal calprotectin cutoff ≤183 µg/g	15.00 (1.33-170.00)	**.029** ^**a**^	—^b^	1.00
**Sigmoid W6**				
Bowel wall thickness (per mm increase)	0.48 (0.24-0.96)	**.038** ^**a**^	0.64 (0.29-1.44)	.28
Bowel wall thickness cutoff ≤3.8 mm	4.50 (0.91-22.15)	.064	1.36 (0.07-28.48)	.84
Color Doppler signal (per 1 category increase)	0.35 (0.14-0.88)	**.026** ^**a**^	0.35 (0.14-0.88)	**.026** ^**a**^
Loss of stratification	0.61 (0.09-4.37)	.62	5.99 (0.33-110.00)	.23
Loss of haustration	0.15 (0.03-0.81)	**.028** ^**a**^	0.52 (0.03-8.57)	.65
Presence of fatty wrapping	0.38 (0.06-2.52)	.31	7.47 (0.29-194.00)	.23
Presence of lymph nodes	—^b^	1.00	—^b^	1.00
Submucosal wall thickness (per mm increase)	0.14 (0.03-0.75)	**.02** ^**a**^	0.21 (0.04-1.04)	.06
Fecal calprotectin cutoff ≤530 µg/g	33.00 (2.91-374.00)	**.005** ^**a**^	11.24 (0.60-210.00)	.11
**Sigmoid W8-W26**				
Bowel wall thickness (per mm increase)	0.07 (0.01-0.47)	**.006** ^**a**^	0.30 (0.03-3.39)	.33
Bowel wall thickness cutoff ≤3.5 mm	91.00 (7.35-1126)	**<.0001** ^**a**^	74.00 (4.72-1159)	**.002** ^**a**^
Color Doppler signal (per 1 category increase)	0.32 (0.13-0.77)	**.012** ^**a**^	0.34 (0.07-1.58)	.17
Loss of stratification	0.33 (0.03-3.64)	.37	—^b^	1.00
Loss of haustration	0.005 (0.00-0.09)	**<.0001** ^**a**^	0.04 (0.001-3.03)	.15
Presence of fatty wrapping	—^b^	1.00	—^b^	1.00
Presence of lymph nodes	—^b^	1.00	—^b^	1.00
Submucosal wall thickness (per mm increase)	0.11 (0.03-0.46)	**.002** ^**a**^	13.05 (0.19-919.00)	.24
Fecal calprotectin cutoff ≤253 µg/g	45.00 (3.47-584.00)	**.004** ^**a**^	—^b^	1.00

Multivariable logistic regression analysis was performed using a backward selection procedure separately for bowel wall thickness as continuous variable and for the defined cutoff values in this manuscript.

Abbreviations: CI, confidence interval; OR, odds ratio; W, week.

^a^ *P* < .05.

^b^When this intestinal ultrasound feature was present, none of the patients reached (W6) or had (W8-W26) endoscopic remission and endoscopic improvement, respectively.

### Endoscopic Response and Improvement of Other IUS Parameters

CDS normalized (CDS ≤1) in patients with endoscopic response in the sigmoid colon from W2 onward and for the descending colon from W6 onward (Supplementary Figure 5), while for endoscopic nonresponders this was not seen. For the other IUS parameters, loss of haustrations, presence of fat wrapping, and presence of lymph nodes significantly improved when there was endoscopic response from W6 onward both in the sigmoid and descending colon (Supplementary Tables 7 and 8).

### Responsiveness of IUS Parameters

**Figure 2.**
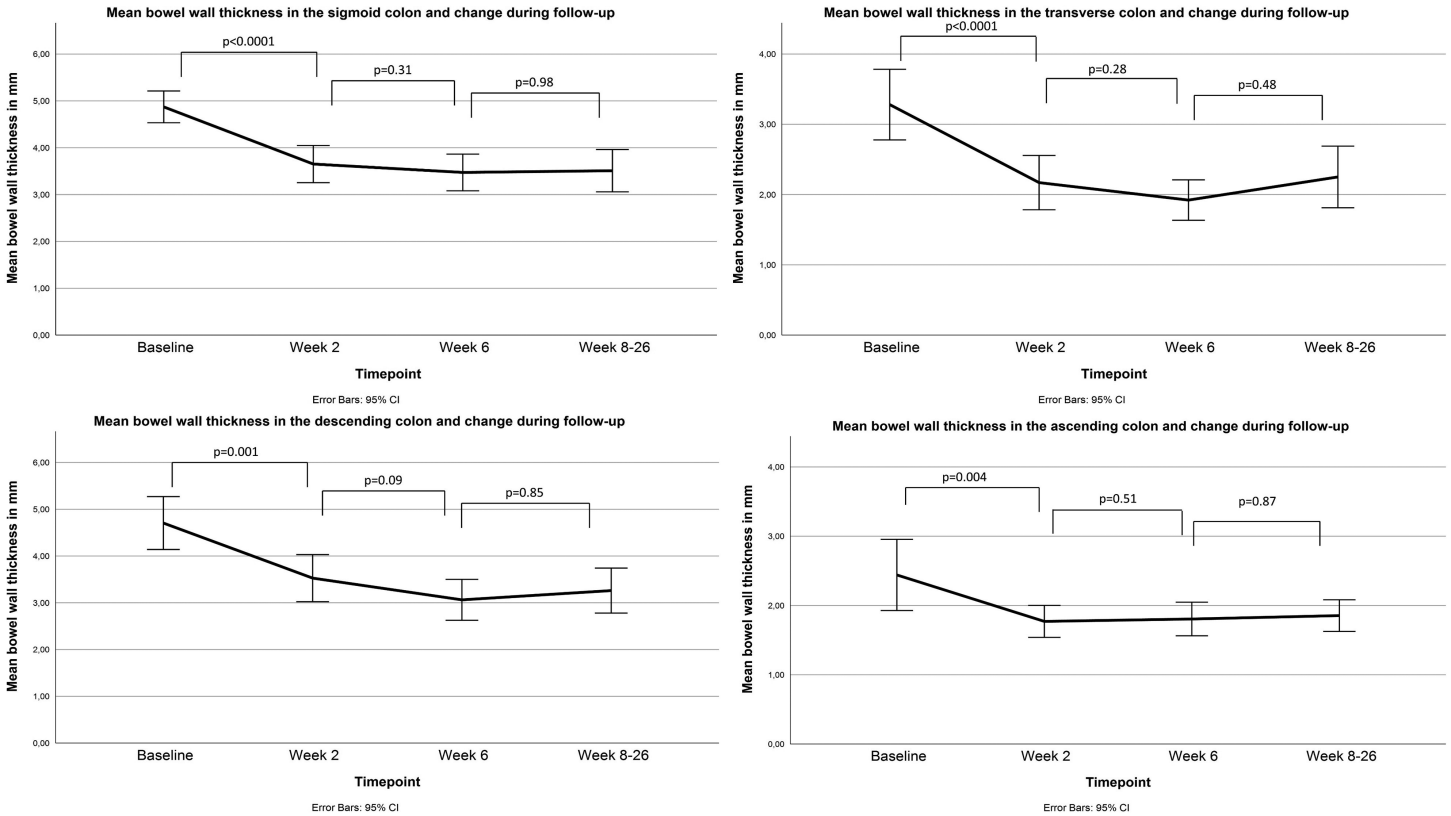
Decrease in bowel wall thickness in the sigmoid, descending, transverse, and ascending colon for the total population. CI, confidence interval.

**Figure 3.**
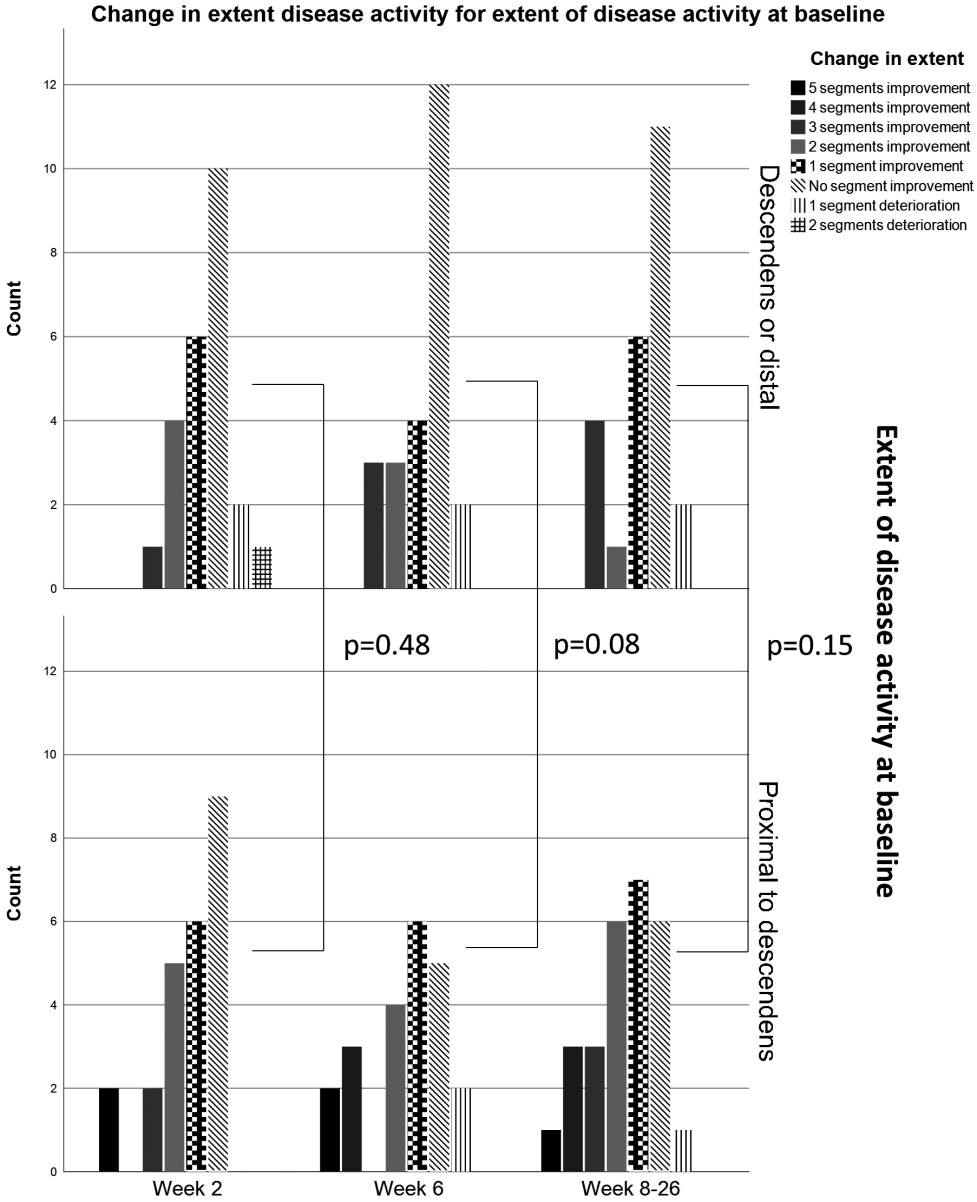
Segmental healing during follow-up for patients with a left-sided colitis (≤ descending colon, n = 24) and patients with an extensive colitis (≥ transverse colon, n = 27) at baseline intestinal ultrasound. Data were analyzed with chi-square test for more than 2 groups.

For the total cohort (n = 51), BWT decreased significantly in all colonic segments (all *P* < .005) from baseline to W2 (Figure 2; Supplementary Table 9). Most significant improvement for the other IUS parameters was between W2 and W6 (Supplementary Figure 6). In addition, most patients showed 1 or 2 segments improvement at W2 whereas at W6 and W8 to W26 disease activity retracted further with >2 segments improvement (Figure 3). This segmental healing was independent of baseline disease extent or Montreal classification.

### Responsiveness of BWT per Clinical Target

For clinical remission according to the Lichtiger score at W8 to W26, BWT was significantly lower from W6 onward compared with patients without clinical remission (3.01  ± 1.34 mm vs 3.85  ± 1.20 mm; *P* = .035). For the SCCAI, BWT was significantly different at W8 to W26 between patients in clinical remission and without clinical remission (2.8  ± 1.1 mm vs 4.2  ± 1.6 mm; *P* = .002) but not at W6 (*P* = .22). Furthermore, decrease in percentage BWT was significantly more pronounced in clinical responders compared with nonresponders for both the SCCAI and Lichtiger score (Supplementary Figure 7). In addition, BWT in distal colonic segments showed higher correlation with clinical scores than the more proximal colonic segments (Supplementary Tables 10 and 11).

### Drug-Specific Response

**Figure 4.**
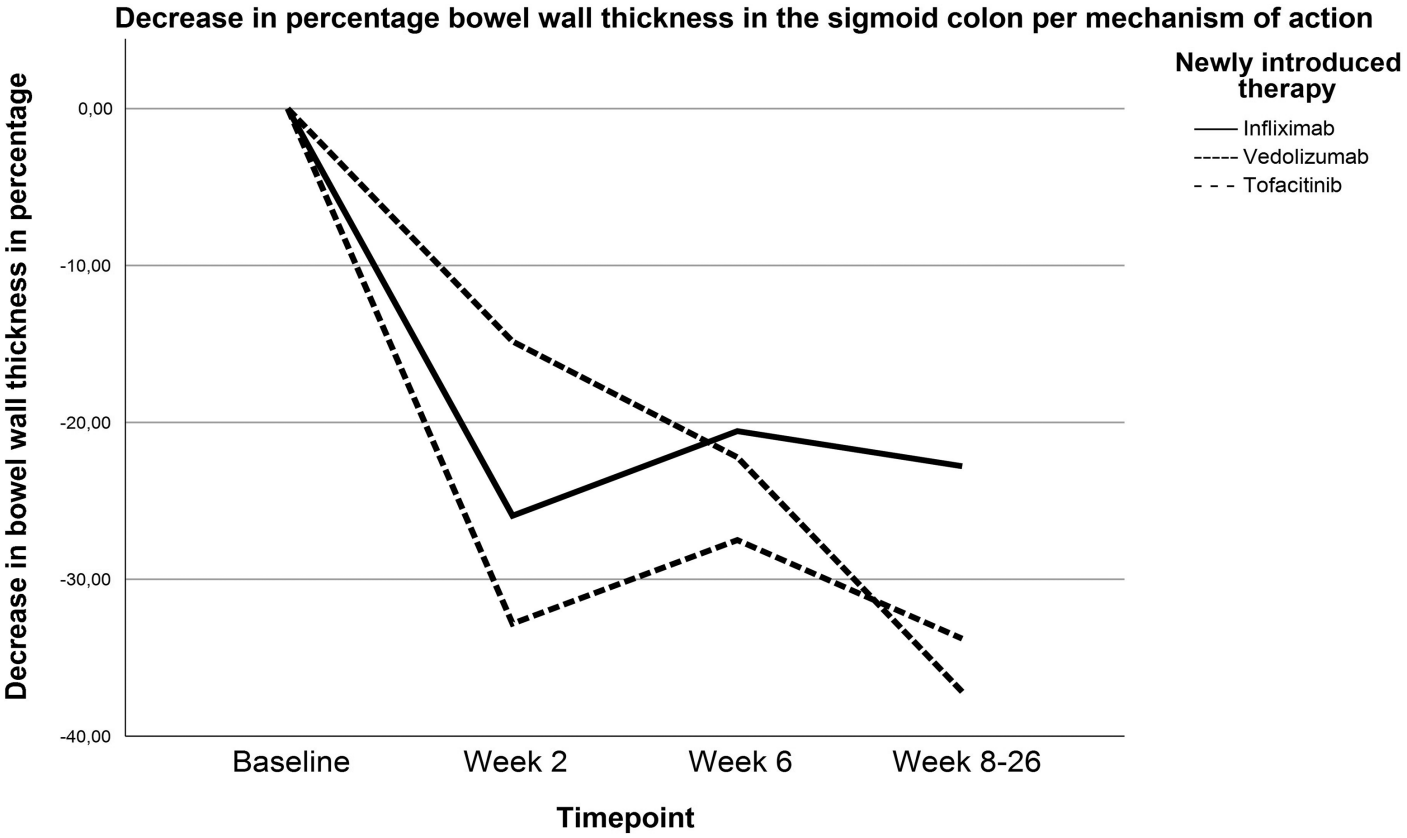
Decrease in median percentage bowel wall thickness with regard to baseline regardless of endoscopic response for patients treated with infliximab (n = 18, week 2 [W2]: −26% [interquartile range [IQR], −43% to −6%], *P* = .001; W6: −23% [IQR, −50% to 4%], *P* = .019; W8-W26: −23% [IQR, −58 to 0%], *P* = .009), vedolizumab (n = 14, W2: −14% [IQR, −43 to 5%], *P* = .11; W6: −22% [IQR, −52% to −16% ], *P* = .04; W8-W26: −37% [IQR, −57% to −17% ], *P* = .008), and tofacitinib (n = 14, W2: −33% [IQR, −46% to −5% ], *P* = .007; W6: −28% [IQR, −38% to −0.5% ], *P* = .028; W8-W26: −33% [IQR, −50% to −2% ], *P* = .013).

For infliximab (n = 18), vedolizumab (n = 14), and tofacitinib (n = 14) sufficient data were available to investigate drug-specific IUS response. Patients treated with infliximab (−26% [IQR, −43% to −6%], *P* = .001) or tofacitinib (−33% [IQR, −46% to −5%], *P* = .007) showed a significant decrease in sigmoid BWT already at W2 as compared with baseline. In comparison, patients treated with vedolizumab did not show a significant decrease at W2 (−14% [IQR, −43% to 5%], *P* = .11) but did as of W6 (−22% [IQR, −52% to −16%; *P* = .04) compared with baseline (Figure 4). By W8 to W26, there was no significant difference among different compounds in terms of change in BWT compared with baseline (Figure 4). Patients treated with infliximab were more frequently biological naive than patients treated with vedolizumab or tofacitinib (72% vs 53% vs 54%; *P* = .019). Patients treated with infliximab were also more frequently on systemic corticosteroids than patients receiving tofacitinib (94% vs 50%; *P* = .004) between vedolizumab-treated and infliximab-treated (64% vs 94%; *P* = .08) or vedolizumab-treated and tofacitinib-treated (64% vs 50%; *P* = .21) patients. Patients on systemic corticosteroids had less decrease of BWT at W2 compared with patients not receiving corticosteroids (−17 ± 26% vs −37 ± 20%; *P* = .023). At W6 and W8 to W26, there was no significant difference anymore (baseline to W6: −24 ± 30% vs −25 ± 27%; *P* = .92; baseline to W8 to W26: −23 ± 31% vs −31 ± 29%; *P* = .46).

### Individual Wall Layer Assessment in the Sigmoid Colon

At baseline the submucosa (2.34  ± 1.03 mm) was significantly thicker than the mucosa (1.16  ± 0.61 mm; *P* < .0001) and muscularis propria (1.28  ± 0.48 mm; *P* < .0001), with no significant difference between mucosa and muscularis propria (*P* = .20). At W8 to W26, the mean submucosa decreased to 0.70 ± 0.32 mm when there was endoscopic remission and there was no difference between the mucosa (0.48 ± 0.19 mm; *P* = .06) and muscularis propria (0.52 ± 0.35 mm; *P* = .33) anymore. At W6, the submucosa was the only wall layer that accurately predicted endoscopic improvement (per mm: OR, 0.14; 95% CI, 0.03-0.75; *P* = .02) and endoscopic remission (per mm: OR, 0.09; 95% CI, 0.01-0.65; *P* = .018). Submucosal thickness at W6 was just not significant (*P* = .06) (Table 3) in multivariate analysis to predict endoscopic improvement in addition to CDS. Interestingly, for endoscopic responders decrease in percentage of the mucosa was more pronounced at W8 to W26 than in nonresponders (−38 ± 36% vs 15 ± 65%; *P* = .014). For the submucosa, this decrease was not significant (−27 ± 53% vs 11 ± 62%; *P* = .09). Endoscopic response could not be predicted with individual wall layer assessment. Furthermore, percentage wall layer of total BWT and ratios between wall layers were analyzed, but these were inferior to absolute measurements.

### Reliability Analysis

For BWT, a strong agreement (intraclass correlation coefficient, 0.90; *P* < .0001) was reached. For pathologic CDS (CDS: 2 or 3) vs nonpathologic CDS (CDS: 0 or 1), the agreement was perfect (κ = 0.90; *P* < .0001). For the other parameters agreement is reported in Supplementary Table 12.

## Discussion

In this prospective, longitudinal cohort of moderate-to-severe UC patients, we have demonstrated that BWT <3.0 mm as measured in the sigmoid 6 weeks after starting anti-inflammatory treatment is accurate to predict endoscopic remission upon follow-up. Improvement in CDS after 6 weeks of treatment predicted endoscopic improvement in the sigmoid. The kinetics of IUS response differed among drugs of different mechanism of action: in patients treated with infliximab and tofacitinib, reduction in BWT was already significant after 2 weeks of treatment, while in vedolizumab-treated patients it became apparent 6 weeks into treatment. However, at further follow-up, no significant difference was detectable among patients receiving different treatments. We have shown that IUS changes, importantly reduction in BWT as a single most important parameter, occur already after 2 weeks of treatment initiation and that the most responsive bowel layer is the submucosa, also when evaluated against endoscopic outcomes. To determine endoscopic remission and improvement, 2.7 mm and 3.5 mm were the best cutoff values for BWT in the sigmoid colon, respectively.

The STRIDE II (Selecting Therapeutic Targets in Inflammatory Bowel Disease II) consensus suggests endoscopic remission as a long-term target after starting anti-inflammatory treatment.^3^ Ideally, treatment response is evaluated with objective measures after treatment initiation, as nonresponse is associated with poor long-term outcomes such as risk for colectomy.^3,25-27^ IUS is a noninvasive tool, which was reported to be sensitive to change already after 2 weeks of anti-inflammatory treatment in patients with UC when compared with clinical outcomes.^11^ We found similar results when treatment response was evaluated against endoscopic outcomes upon follow-up and when stratified for endpoints, from 6 weeks onward BWT was significantly different between patients with or without endoscopic remission or improvement.

Indeed, BWT has previously shown to be a single most important parameter to determine endoscopic remission.^9,10,24^ Previous studies have debated the most optimal cutoff values for BWT to detect disease flares. A recent systematic review suggested 4.0 mm as most accurate cutoff value to distinguish active from quiescent disease with endoscopy as gold standard.^14^ However, this cutoff value was not specific for a particular colonic segment (ie, sigmoid or descending colon), and there was a large heterogeneity among endoscopic scoring systems and definitions for endoscopic remission/improvement.^14^ Previous cross-sectional studies found ≤3.0 mm as the ideal cutoff to identify EMS ≤1 independent of colonic segment.^9,10^ This is close to our 3.5-mm cutoff value for the sigmoid colon, and the slight discrepancy could be explained by the differences in study design: while other studies were cross-sectional, in the present study response was assessed in a longitudinal follow-up, in which BWT might have decreased further after W8 to W26, especially in those patients who were evaluated early in the W8 to W26 time frame.

As endoscopic remission is associated with improved long-term outcomes, IUS should ideally be able to distinguish between endoscopic remission and endoscopic improvement.^28^ As compared with a previous cross-sectional cohort in which a cutoff of 2.1 mm was found to determine endoscopic remission, we found 2.7 mm as an accurate cutoff value.^10^ In a univariate analysis, we also found CDS and loss of haustrations being discriminative for endoscopic improvement but not for endoscopic remission. As we had limited patient numbers for reaching endoscopic improvement but no endoscopic remission, we could not assess the discriminative value for CDS and loss of haustrations to distinguish endoscopic remission and endoscopic improvement. In that perspective, Allocca et al^9^ and Bots et al^10^ developed a promising scoring index for UC incorporating BWT, CDS, loss of haustrations, and fatty wrapping. Whether these scoring indices are accurate to determine treatment response and distinguishes endoscopic remission from improvement is currently under investigation.

Based on an expert consensus, treatment response has been defined as a decrease of 25% in BWT or decrease of >1.0 mm with decrease in ≥1 CDS category ideally evaluated after 14 weeks.^13^ In our cohort, we indeed demonstrated a decrease of 23% and 40% in BWT after 18 weeks as most accurate for the sigmoid and descending colon to determine endoscopic response, respectively. With regard to clinical response, Maaser et al found a 34% and 35% decrease in BWT for the sigmoid and descending colon, respectively, in responders but did not evaluate BWT against an endoscopic reference standard.^11^ Furthermore, improvement in ≥1 CDS category, return of haustrations, normalization of fatty wrapping or normalization of lymph nodes were associated with endoscopic response. In patients with a borderline decrease in BWT, these additional IUS parameters might increase accuracy to determine response to treatment.

In our cohort, 18 patients did not undergo a second endoscopy, predominantly due to the COVID-19 pandemic (n = 10). A total of 8 of these patients had presence of disease activity at IUS, with an FCP ≥250 µg/g, and treatment was escalated (n = 8), whereas the other 2 patients had normal IUS findings, an FCP <250 µg/g, and clinical remission (SCCAI ≤2 and Lichtiger ≤3). In addition, 4 patients refused a second endoscopy because they were in clinical remission with normal IUS findings. Although forced due to the pandemic, this may demonstrate the application for IUS as point-of-care tool in a close monitoring setting, thereby guiding treatment decision making. On the other hand, in the current study, the absence of a second endoscopy created a selection bias, with respectively a higher percentage of patients responding to therapy receiving a second endoscopy. Hence, the 40% decrease in BWT for endoscopic responders might be too rigorous as cutoff value.

Interestingly, we demonstrated that the kinetics of IUS response was drug specific, and these findings are in line with a previous Delphi consensus.^3^ Although patients in the vedolizumab group were less frequently biological naive when compared with the infliximab group, there was no difference in bio-exposure between the vedolizumab and tofacitinib group; hence bio-naivety could potentially be a confounder explaining the difference for decrease in BWT at W2. This is in contrast to corticosteroid use, which could not be held responsible for the difference in early response among mechanisms of action, as corticosteroid use was associated with less decrease of BWT at W2. As more than half of the patients that did not receive corticosteroids were treated with tofacitinib, the rapid response in the noncorticosteroid group could also be explained by the use of tofacitinib.

The STRIDE II consensus suggests clinical, biochemical, and endoscopic treatment targets as immediate, short-term, intermediate-term, or long-term targets.^3^ With the high accuracy of early IUS to predict endoscopic outcomes, IUS has the potential to become an additional treatment target in the future. Early, noninvasive evaluation and accurate detection of need for treatment optimization could potentially offer improved long-term outcomes in patients with UC. Interestingly, we found the submucosa to be the most responsive wall layer, and recent studies also demonstrated that the submucosa was the most affected in colectomy resection specimens.^24,29,30^ Consequently, a transmural target for UC might have potential in treatment follow-up if these findings are confirmed.

In UC, a segmental healing pattern has been observed previously.^31-33^ In agreement, in our cohort, pathologic bowel segments started to normalize at the most proximally affected segment and subsequent distal segments followed over time. Recently, endoscopic scoring indices have been developed to assess disease activity and severity per segment^34,35^ instead of commonly used indices that assess response in the worst affected segment.^36-38^ A segmental approach takes disease extent and overall inflammatory burden into consideration. IUS has the advantage to visualize all segments in a noninvasive manner when assessing treatment response and is therefore of additional value to other noninvasive alternatives to endoscopy such as FCP.

Our study has its limitations. First, limited number of patients underwent complete colonoscopy at follow-up W8 to W26; hence, endoscopic data on the transverse and ascending colon were limited. Furthermore, the 8- to 26-week time frame is wide for an endpoint in a prospective design. The COVID-19 pandemic put significant pressure on our endoscopy program and endoscopies were performed less frequently or postponed. However, we have demonstrated that decrease in BWT was not significantly different between patients undergoing endoscopy between W8 and W17 vs W18 and W26. Therefore, bias as a consequence of the wide time frame is probably limited. Although we hypothesized that a segmental approach is important, assessment of proximal segments was therefore often performed without endoscopic reference standard. Second, we assessed changes after starting anti-inflammatory treatment of different mechanism of action, which could have resulted in heterogeneity. However, sample sizes were sufficient to demonstrate differences among 3 treatments with different modes of action. Last, we used the EMS to evaluate endoscopic outcomes, which may not be the most sensitive score to detect endoscopic response.^33,39,40^ Therefore, it would of future interest to compare responsiveness of BWT with other endoscopic scores, such as the Ulcerative Colitis Endoscopic Index of Severity.

Our study also has several advantages. We had a prospective design with close follow-up, powered the study beforehand, and used endoscopy as reference standard to analyze segmental changes. Besides endoscopic targets, we also analyzed commonly used clinical and biochemical outcomes and demonstrated correlation between IUS and these parameters. Furthermore, we performed a reliability analysis, and our findings were in line with previous results.^9,10,12^

Future studies should confirm our results and proposed cutoff values for BWT. In addition, the existing IUS scores^9,10^ need complete validation for distinguishing active from inactive disease, severity of disease, response to treatment, and segmental healing, ideally for multiple mechanisms of action. Moreover, including clinical scores and biochemical parameters could provide a noninvasive scoring index. Additionally, further evaluation of the submucosal layer in UC is of interest for future research.

## Conclusions

We have demonstrated that early IUS at 6 weeks after treatment initiation is accurate to predict endoscopic remission and improvement in patients with moderate-to-severe UC and that the time kinetic of IUS changes is drug specific. IUS is accurate for close monitoring in the treatment in UC and is a surrogate marker of endoscopy.

## Supplementary data

Supplementary data is available at *Inflammatory Bowel Diseases* online.

izad274_suppl_Supplementary_Material

## Data Availability

The data underlying this article will be shared on reasonable request to the corresponding author.
