# Supplementary material for: Early Intestinal Ultrasound Predicts Clinical and Endoscopic Treatment Response and Demonstrates Drug-Specific Kinetics in Moderate-to-Severe Ulcerative Colitis
Source: Inflamm Bowel Dis. 2023 Nov 27;30(11):1992–2003. doi: 10.1093/ibd/izad274 (PMC11532594; doi:10.1093/ibd/izad274)
Supplement: izad274_suppl_Supplementary_Material [file izad274_suppl_supplementary_material.docx]

SUPPLEMENTARY

| **Endoscopic target** | Baseline (mean±SD) | Week 2  (mean±SD) | Week 6  (mean±SD) | Week 8-26  (mean±SD) |
| --- | --- | --- | --- | --- |
| **Endoscopic remission** | 4.94 ± 1.77 | 3.28 ± 1.61 | 2.51 ± 1.18 | 1.88 ± 0.49 |
| **No endoscopic remission** | 5.00 ± 1.12 | 3.89 ± 1.22 | 4.07 ± 1.11 | 4.15 ± 1.51 |
| **p-value** | 0.93 | 0.27 | **0.002** | **<0.0001** |
|  |  |  |  |  |
| **Endoscopic improvement** | 4.78 ± 1.59 | 3.25 ± 1.40 | 3.01 ± 1.21 | 2.29 ± 0.81 |
| **No endoscopic improvement** | 5.16 ± 1.04 | 4.13 ± 1.20 | 4.12 ± 1.26 | 4.64 ± 1.42 |
| **p-value** | 0.42 | 0.08 | **0.026** | **<0.0001** |
|  |  |  |  |  |
| **Endoscopic response** | n.a. | -16% ± 24 | -28% ± 25 | -40% ± 25 |
| **No endoscopic response** | n.a | -23% ± 26 | -9% ± 29 | -4% ± 28 |
| **p-value** | n.a. | 0.42 | 0.09 | **0.001** |

**Supplementary Table 1:** Bowel wall thickness in mm per time-point for patients with and without endoscopic improvement and endoscopic remission. Decrease in bowel wall thickness in percentage with regards to baseline (W0) for patients with and without endoscopic response. Data is also represented in Figure 2. [SD: Standard deviation]

| **Sigmoid colon** | Cut-off value | Sensitivity | Specificity | Area under the curve | p-value |
| --- | --- | --- | --- | --- | --- |
| **Endoscopic remission** |  |  |  |  |  |
| Week 2 | 3.4 mm | 71% | 67% | 0.70 [0.47-0.93] | 0.14 |
| Week 6 | 3.0 mm | 78% | 90% | 0.82 [0.63-1.00] | **0.007** |
| Week 8-26 | 2.7 mm | 100% | 86% | 0.95 [0.88-1.00] | **<0.0001** |
| **Endoscopic improvement** |  |  |  |  |  |
| Week 2 | 3.9 mm | 71% | 67% | 0.68 [0.48-0.88] | 0.09 |
| Week 6 | 3.8 mm | 82% | 54% | 0.72 [0.50-0.93] | 0.09 |
| Week 8-26 | 3.5 mm | 91% | 91% | 0.96 [0.88-1.00] | **<0.0001** |
| **Endoscopic response** |  |  |  |  |  |
| ΔBWT W2-W0 in percentage | -13% | 58% | 56% | 0.48 [0.22-0.74] | 0.89 |
| ΔBWT W6-W0 in percentage | -19% | 75% | 67% | 0.67 [0.42-0.91] | 0.20 |
| ΔBWT W8-26-W0 in percentage | -23% | 75% | 89% | 0.81 [0.61-1.00] | **0.019** |

**Supplementary Table 2:** Tables for Supplementary Figure 4 demonstrating accuracy, sensitivity and specificity for bowel wall thickness cut-off values at different time-points to predict/determine endoscopic remission or endoscopic improvement in the sigmoid colon. [BWT: Bowel wall thickness]

| **Descending colon** | **AUROC (95%CI)** | **Cut-off value** | **Sensitivity** | **Specificity** | **p-value** |
| --- | --- | --- | --- | --- | --- |
| **Endoscopic remission** |  |  |  |  |  |
| Week 2 | 0.87 (0.71-1.00) | 3.62 mm | 73% | 89% | **0.006** |
| Week 6 | 0.89 (0.74-1.00) | 3.20 mm | 73% | 100% | **0.003** |
| Week 8-26 | 0.96 (0.87-1.00) | 2.62 mm | 82% | 89% | **0.001** |
| **Endoscopic improvement** |  |  |  |  |  |
| Week 2 | 0.63 (0.37-0.88) | 4.56 mm | 77% | 57% | 0.36 |
| Week 6 | 0.69 (0.44-0.94) | 3.49 mm | 77% | 57% | 0.17 |
| Week 8-26 | 0.76 (0.54-0.97) | 2.61 mm | 69% | 86% | 0.06 |
| **Endoscopic response** |  |  |  |  |  |
| ΔBWT W2-W0 in percentage | 0.69 (0.45-0.93) | -13% | 70% | 56% | 0.17 |
| ΔBWT W6-W0 in percentage | 0.64 (0.39-0.90) | -19% | 60% | 56% | 0.39 |
| ΔBWT W8-26-W0 in percentage | 0.74 (0.51-0.98) | -39% | 100% | 56% | 0.07 |

**Supplementary Table 3:** Tables demonstrating accuracy, sensitivity and specificity for bowel wall thickness cut-off values at different time-points to predict/determine endoscopic remission, endoscopic improvement or endoscopic response in the descending colon. [BWT: Bowel wall thickness]

| **Sigmoid colon** | **AUROC (95%CI)** | **Cut-off value** | **Sensitivity** | **Specificity** | **PPV/NPV** | **p-value** |
| --- | --- | --- | --- | --- | --- | --- |
| **Endoscopic remission** |  |  |  |  |  |  |
| Week 2 | 0.61 (0.35-0.87) | 762 µg/g | 83% | 43% | n.a. | 0.46 |
| Week 6 | 0.74 (0.46-1.00) | 626 µg/g | 83% | 71% | n.a. | 0.10 |
| Week 8-26 | 0.88 (0.72-1.00) | 183 µg/g | 83% | 75% | 56%/92% | **0.008** |
| **Endoscopic improvement** |  |  |  |  |  |  |
| Week 2 | 0.63 (0.38-0.89) | 762 µg/g | 78% | 64% | n.a. | 0.31 |
| Week 6 | 0.78 (0.52-1.00) | 530 µg/g | 78% | 91% | 90%/83% | **0.03** |
| Week 8-26 | 0.88 (0.71-1.00) | 253 µg/g | 82% | 91% | 90%/79% | **0.003** |
| **Endoscopic response** |  |  |  |  |  |  |
| ΔBWT W2-W0 in percentage | 0.69 (0.39-0.99) | -78% | 50% | 86% | n.a. | 0.25 |
| ΔBWT W6-W0 in percentage | 0.67 (0.33-1.00) | -39% | 67% | 86% | n.a. | 0.32 |
| ΔBWT W8-26-W0 in percentage | 0.79 (0.52-1.00) | -86% | 50% | 100% | n.a. | 0.09 |

**Supplementary Table 4:** Tables demonstrating accuracy, sensitivity and specificity for fecal calprotectin cut-off values at different time-points to predict/determine endoscopic remission, endoscopic improvement or endoscopic response in the sigmoid colon.

| **Logistic regression for endoscopic improvement** | Univariable | | Multivariable | |
| --- | --- | --- | --- | --- |
| **Descending W6** | Odds-ratio | p-value | Odds-ratio | p-value |
|  |  | |  | |
| Bowel wall thickness cut-off ≤3.5 mm | 2.29 [0.44-11.92] | 0.33 | 1.71 [0.29-10.03] | 0.56 |
| Colour Doppler Signal (per Limberg category increase) | 0.22 [0.06-0.74] | **0.015** | 0.22 [0.06-0.74] | **0.015** |
| Loss of stratification | 0.09 [0.02-2.02] | 0.20 | 0.22 [0.01-3.50] | 0.28 |
| Loss of haustration | 0.27 [0.05-1.50] | 0.14 | 0.99 [0.01-9.88] | 1.00 |
| Presence of fatty wrapping | ^a^ | 1.00 | ^a^ | 1.00 |
| Presence of lymph nodes | 0.47 [0.05-4.03] | 0.49 | 0.01 [0.00-5.13] | 0.15 |
| **Descendens W8-26** |  |  |  |  |
| Bowel wall thickness (per mm increase) | 0.48 [0.27-0.86] | **0.014** | 1.02 [0.39-2.67] | 0.97 |
| Bowel wall thickness cut-off ≤2.6 mm | 18.33 [1.87-180] | **0.013** | 18.33 [1.87-180] | **0.013** |
| Colour Doppler Signal (per Limberg category increase) | 0.43 [0.18-1.02] | 0.055 | 0.69 [0.27-1.78] | 0.44 |
| Loss of stratification | ^a^ | 1.00 | ^a^ | 1.00 |
| Loss of haustration | 0.09 [0.015-0.59] | **0.012** | 0.06 [0.006-0.72] | **0.026** |
| Presence of fatty wrapping | ^a^ | 1.00 | ^a^ | 1.00 |
| Presence of lymph nodes | 0.23 [0.034-1.60] | 0.14 | 0.84 [0.09-8.18] | 0.88 |

**Supplementary Table 5:** Univariable and multivariable logistic regression analysis to predict endoscopic improvement at week 6 (W6) and week 8-26 (W8-26) in the descending colon. Multivariable logistic regression analysis was performed using a backward selection procedure separately for bowel wall thickness as continuous variable and for the defined cut-off values in this manuscript. [^a^: when this IUS feature was present, none of the patients reached (W6) or had (W8-26) endoscopic remission and endoscopic improvement, respectively]

|  | ΔBWT W0-W2 | ΔBWT W0-W6 | ΔBWT W0-W8-26 |
| --- | --- | --- | --- |
| ΔBWT per percentage decrease | p=0.26 | p=1.00 | **OR: 1.02 [1.00-1.05)**  **p=0.47** |
| Improvement in CDS≥1 category | p=0.49 | p=0.06 | **OR: 11.00 [1.60-75.50], p=0.015** |
| Normalization of haustrations | p=0.94 | p=0.62 | p=0.62 |
| Normalization of WLS | p=1.00 | p=0.99 | p=0.99 |
| Normalization of LN | p=0.81 | p=0.83 | p=0.83 |
| Normalization of FW | p=0.97 | p=0.06 | p=0.06 |

**Supplementary Table 6:** Multivariable logistic regression analysis to predict endoscopic response at week 2 (W2), week 6 (W6) and week 8-26 (W8-26) in the descending colon.

| Sigmoid colon IUS parameters | **Baseline**  *(n=33)* | **Week 2**  *(n=29)* | **Week 6**  *(n=33)* | **Week 8-26**  *(n=33)* | **ΔW0-W2**  *(n=29)* | **ΔW0-W6**  *(n=33)* | **ΔW0-W8-26**  *(n=33)* |
| --- | --- | --- | --- | --- | --- | --- | --- |
| **Loss of WLS**   - Endoscopic response W8-26 - Endoscopic   non-response W8-26 | 3 (9%)  6 (18%) | 3 (10%)  4 (14%) | 1 (4%)  5 (15%) | 3 (10%)  1 (3%) | p=ns  p=ns | p=ns  p=ns | p=ns  p=ns |
| **Loss of haustrations**   - Endoscopic response W8-26 - Endoscopic   non-response W8-26 | 18 (55%)  12 (36%) | 11 (38%)  9 (31%) | 5 (15%)  9 (30%) | 5 (14%)  9 (28%) | p=ns  p=ns | **p=0.001**  p=ns | **p=0.001**  p=ns |
| **Presence of FW**   - Endoscopic response W8-26 - Endoscopic   non-response W8-26 | 13 (39%)  7 (21%) | 7 (24%)  1 (3%) | 4 (11%)  4 (11%) | 0 (0%)  6 (17%) | p=ns  p=ns | **p=0.004**  p=ns | **p<0.0001**  p=ns |
| **Presence of LN**   - Endoscopic response W8-26 - Endoscopic   non-response W8-26 | 11 (33%)  10 (30%) | 3 (10%)  4 (14%) | 4 (11%)  4 (11%) | 2 (7%)  10 (31%) | **p=0.03**  p=ns | **p=0.03**  p=ns | **p=0.01**  p=ns |

**Supplementary Table 7:** Change for loss of wall layer stratification, loss of haustrations, presence of fatty wrapping and presence of lymph nodes in patients with and without endoscopic response in the sigmoid colon analyzed with paired McNemar Test. [WLS: wall layer stratification; FW: fatty wrapping; LN: lymph nodes; W0: baseline; W2: week 2; W6: week 6; W8-26: week 8-26]

| Descending colon IUS parameters | **Baseline**  *(n=31)* | **Week 2**  *(n=27)* | **Week 6**  *(n=30)* | **Week 8-26**  *(n=31)* | **ΔW0-W2**  *(n=27)* | **ΔW0-W6**  *(n=30)* | **ΔW0-W8-26**  *(n=31)* |
| --- | --- | --- | --- | --- | --- | --- | --- |
| **Loss of WLS**   - Endoscopic response W8-26 - Endoscopic   non-response W8-26 | 5 (16%)  4 (13%) | 5 (19%)  4 (15%) | 2 (7%)  4 (13%) | 0 (0%)  4 (13%) | p=ns  p=ns | p=ns  p=ns | **p<0.0001**  p=ns |
| **Loss of haustrations**   - Endoscopic response W8-26 - Endoscopic   non-response W8-26 | 14 (48%)  10 (32%) | 8 (30%)  9 (33%) | 7 (24%)  7 (24%) | 5 (16%)  11 (35%) | p=ns  p=ns | **p=0.03**  p=ns | **p=0.02**  p=ns |
| **Presence of FW**   - Endoscopic response W8-26 - Endoscopic   non-response W8-26 | 9 (31%)  7 (24%) | 3 (11%)  3 (11%) | 0 (0%)  4 (13%) | 0 (0%)  4 (13%) | p=ns  p=ns | **P<0.0001**  p=ns | **p<0.0001**  p=ns |
| **Presence of LN**   - Endoscopic response W8-26 - Endoscopic   non-response W8-26 | 9 (29%)  7 (23%) | 2 (7%)  3 (11%) | 2 (7%)  2 (7%) | 2 (6%)  5 (16%) | p=ns  p=ns | **p=0.03**  p=ns | **p=0.04**  p=ns |

**Supplementary Table 8:** Change for loss of wall layer stratification, loss of haustrations, presence of fatty wrapping and presence of lymph nodes in patients with and without endoscopic response in the descending colon analyzed with a paired McNemar Test. [WLS: wall layer stratification; FW: fatty wrapping; LN: lymph nodes; W0: baseline; W2: week 2; W6: week 6; W8-26: week 8-26]

| Colonic segment | BWT W0 | BWT W2 | BWT W6 | BWT W8-26 | p-value |
| --- | --- | --- | --- | --- | --- |
| Sigmoid colon  ΔW0-W2 (n=47)  ΔW2-W6 (n=43)  ΔW6-W8-26 (n=41) | 4.8 ± 1.1 | 3.7 ± 1.4 | 3.4 ± 1.3 | 3.4 ± 1.4 |  |
|  |  | | | | **<0.0001** |
|  |  |  |  |  | 0.31 |
|  |  |  |  |  | 0.98 |
| Descending colon  ΔW0-W2 (n=47)  ΔW2-W6 (n=43)  ΔW6-W8-26 (n=41) | 4.5 ± 1.8 | 3.5 ± 1.7 | 2.9 ± 1.4 | 3.1 ± 1.5 |  |
|  |  | | | | **0.001** |
|  |  |  |  |  | 0.09 |
|  |  |  |  |  | 0.85 |
| Transverse colon  ΔW0-W2 (n=47)  ΔW2-W6 (n=43)  ΔW6-W8-26 (n=41) | 3.2 ± 1.9 | 2.2 ± 1.3 | 2.0 ± 1.2 | 2.1 ± 1.2 |  |
|  |  | | | | **<0.0001** |
|  |  |  |  |  | 0.28 |
|  |  |  |  |  | 0.48 |
| Ascending colon  ΔW0-W2 (n=46)  ΔW2-W6 (n=43)  ΔW6-W8-26 (n=42) | 2.5 ± 1.9 | 1.8 ± 0.8 | 1.8 ± 0.8 | 1.9 ± 0.8 |  |
|  |  | | | | **0.004** |
|  |  |  |  |  | 0.51 |
|  |  |  |  |  | 0.87 |

**Supplementary Table 9:** Paired analysis for change in bowel wall thickness per colonic segment per time-point regardless of clinical or endoscopic target [BWT=bowel wall thickness)

| Clinical or biochemical parameter | BWT in sigmoid colon | BWT in descending colon | BWT in transverse colon | BWT in ascending colon |
| --- | --- | --- | --- | --- |
| SCCAI score | **ρ=0.63, p<0.0001**  ***n=191*** | **ρ=0.56, p<0.0001**  ***n=191*** | **ρ=0.52, p<0.0001**  ***n=190*** | **ρ=0.33, p=0.0001**  ***n=190*** |
| Lichtiger score | **ρ=0.65, p<0.0001**  ***n=192*** | **ρ=0.59, p<0.0001**  ***n=192*** | **ρ=0.52, p<0.0001**  ***n=191*** | **ρ=0.31, p<0.0001**  ***n=191*** |
| CRP [mg/L] | **ρ=0.41, p<0.0001**  ***n=186*** | **ρ=0.47, p<0.0001**  ***n=186*** | **ρ=0.39, p<0.0001**  ***n=185*** | ρ=0.06, p=0.39  *n=185* |
| Hemoglobin [mmol/L] | **ρ=-0.21, p=0.005**  ***n=184*** | **ρ=-0.31, p=0.005**  ***n=184*** | **ρ=-0.31, p=0.005**  ***n=183*** | ρ=-0.09, p=0.25  *n=183* |
| Leukocyte count [10^9^/L] | **ρ=0.18, p=0.016**  ***n=185*** | **ρ=0.19, p=0.010**  ***n=185*** | ρ=0.09, p=0.24  *n=184* | ρ=0.08, p=0.29  *n=184* |
| Platelet count [10^9^/L] | **ρ=0.32, p<0.0001**  ***n=186*** | **ρ=0.42, p<0.0001**  ***n=186*** | **ρ=0.39, p<0.0001**  ***n=185*** | **ρ=0.23, p=0.001**  ***n=185*** |
| Albumin [g/L] | **ρ=-0.50, p<0.0001**  ***n=175*** | **ρ=-0.55, p<0.0001**  ***n=175*** | **ρ=-0.46, p<0.0001**  ***n=174*** | **ρ=-0.21, p=0.006**  ***n=174*** |
| Fecal calprotectin [µg/g] | **ρ=0.48, p<0.0001**  ***n=150*** | **ρ=0.37, p<0.0001**  ***n=150*** | **ρ=0.26, p=0.001**  ***n=149*** | ρ=0.10, p=0.25  *n=149* |

**Supplementary Table 10:** Correlation for pooled clinical and biochemical parameters with bowel wall thickness per colonic segment. Measurements were performed at baseline (W0), after 2 weeks (W2), 6 weeks (W6) and close to second endoscopy between 8 and 26 weeks (W8-26). [BWT: bowel wall thickness; CRP: C-reactive protein]

| Δ Clinical or biochemical parameter | Δ BWT in sigmoid colon | Δ BWT in descending colon | Δ BWT in transverse colon | Δ BWT in ascending colon |
| --- | --- | --- | --- | --- |
| Δ SCCAI score | **ρ=0.54, p<0.0001**  ***n=46*** | **ρ=0.35, p=0.02**  ***n=46*** | ρ=0.27, p=0.08  *n=45* | ρ=0.04, p=0.80  *n=46* |
| Δ Lichtiger score | **ρ=0.55, p<0.0001**  ***n=46*** | **ρ=0.34, p=0.02**  ***n=46*** | ρ=0.28, p=0.06  *n=45* | ρ=0.01, p=0.97  *n=46* |
| Δ CRP [mg/L] | **ρ=0.34, p=0.02**  ***n=44*** | **ρ=0.42, p=0.005**  ***n=44*** | ρ=0.11, p=0.49  *n=43* | ρ=0.02, p=0.91  *n=43* |
| Δ Hemoglobin [mmol/L] | ρ=0.01, p=0.95  *n=44* | ρ=-0.14, p=0.38  *n=44* | ρ=-0.03, p=0.86  *n=43* | ρ=-0.12, p=0.46  *n=43* |
| Δ Leukocyte count [10^9^/L] | ρ=0.09, p=0.57  *n=43* | ρ=0.28, p=0.08  *n=43* | ρ=0.02, p=0.89  *n=42* | ρ=-0.02, p=0.90  *n=42* |
| Δ Platelet count [10^9^/L] | ρ=0.26, p=0.08  *n=44* | **ρ=0.34, p=0.03**  ***n=44*** | ρ=0.25, p=0.11  *n=43* | ρ=0.21, p=0.18  *n=43* |
| Δ Albumin [g/L] | **ρ=-0.40, p=0.01**  ***n=39*** | **ρ=-0.36, p=0.02**  ***n=39*** | ρ=-0.02, p=0.89  *n=38* | ρ=-0.23, p=0.17  *n=38* |
| Δ Fecal calprotectin [µg/g] | ρ=0.26, p=0.16  *n=30* | ρ=-0.05, p=0.79  *n=30* | ρ=-0.29, p=0.13  *n=29* | ρ=-0.09, p=0.65  *n=30* |

**Supplementary Table 11:** Correlation between Δ bowel wall thickness and Δ for clinical and biochemical parameters between W0 and W8-26. [BWT: bowel wall thickness; CRP: C-reactive protein]

| IUS parameter | Agreement (ICC or κ) | Definition | p-value |
| --- | --- | --- | --- |
| Bowel wall thickness *[continuous in mm]* | ICC: 0.90, 95%CI: 0.79-0.95 | Strong | **<0.0001** |
| Colour Doppler Signal *[pathologic (CDS 2-3) vs non-pathologic (CDS 0-1)]* | κ=0.90, 95%CI: 0.56-1.00 | Perfect | **<0.0001** |
| Colour Doppler Signal *[four CDS categories: 0-3]* | κ=0.27, 95%CI: 0.05-0.50 | Fair | **0.017** |
| Loss of haustrations *[yes/no]* | κ=0.48, 95%CI: 0.10-0.89 | Moderate | **0.005** |
| Loss of stratification *[yes/no]* | κ=0.38, 95%CI: 0.02-0.74 | Fair | **0.04** |
| Presence of fatty wrapping *[yes/no]* | κ=0.33, 95%CI: 0.08-0.59 | Fair | **0.011** |
| Presence of lymph nodes *[yes/no]* | κ=0.31, 95%CI: -0.05-0.67 | Fair | 0.09 |

**Supplementary Table 12:** Inter-observer agreement analysis for all ultrasonographic parameters in the sigmoid colon for thirty randomly selected examinations [CDS=Colour Doppler Signal, ICC=intra-class correlation coefficient, κ=kappa)

**
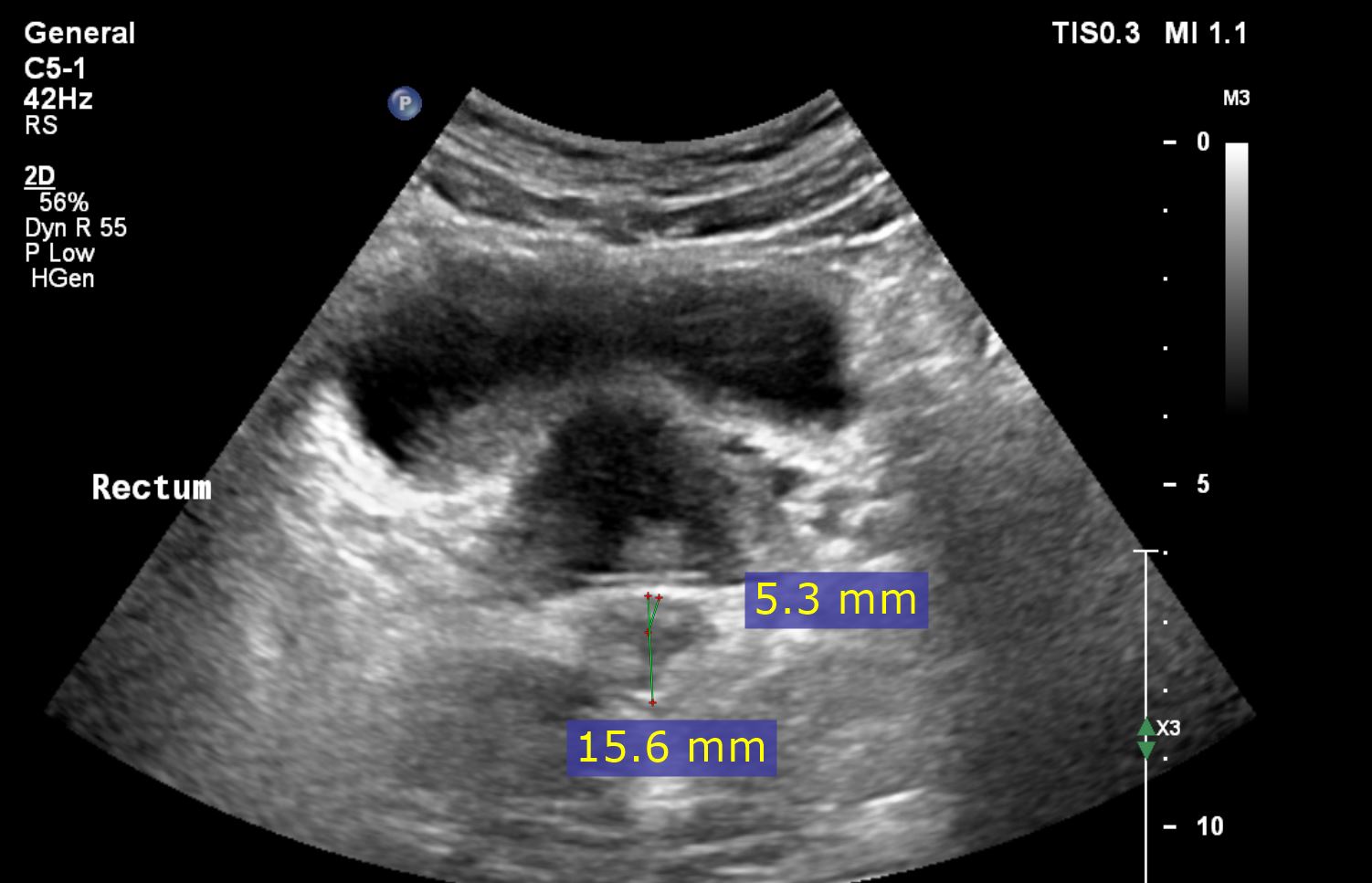
**

**Supplementary Figure 1:** the rectum with 5.3 mm BWT of the anterior wall and 1.56 cm diameter in cross-sectional plane. The upper black cavity is the bladder and the lower black cavity the uterus.

**Supplementary Figure 2:** Consort flow-diagram (presence of IUS disease activity=BWT>3.0 mm and one other pathological parameter in ≥ one segment, normal IUS findings= BWT≤3.0 mm and no other pathological parameter for every segment) [IUS=intestinal ultrasound, W8-26=week 8-26)


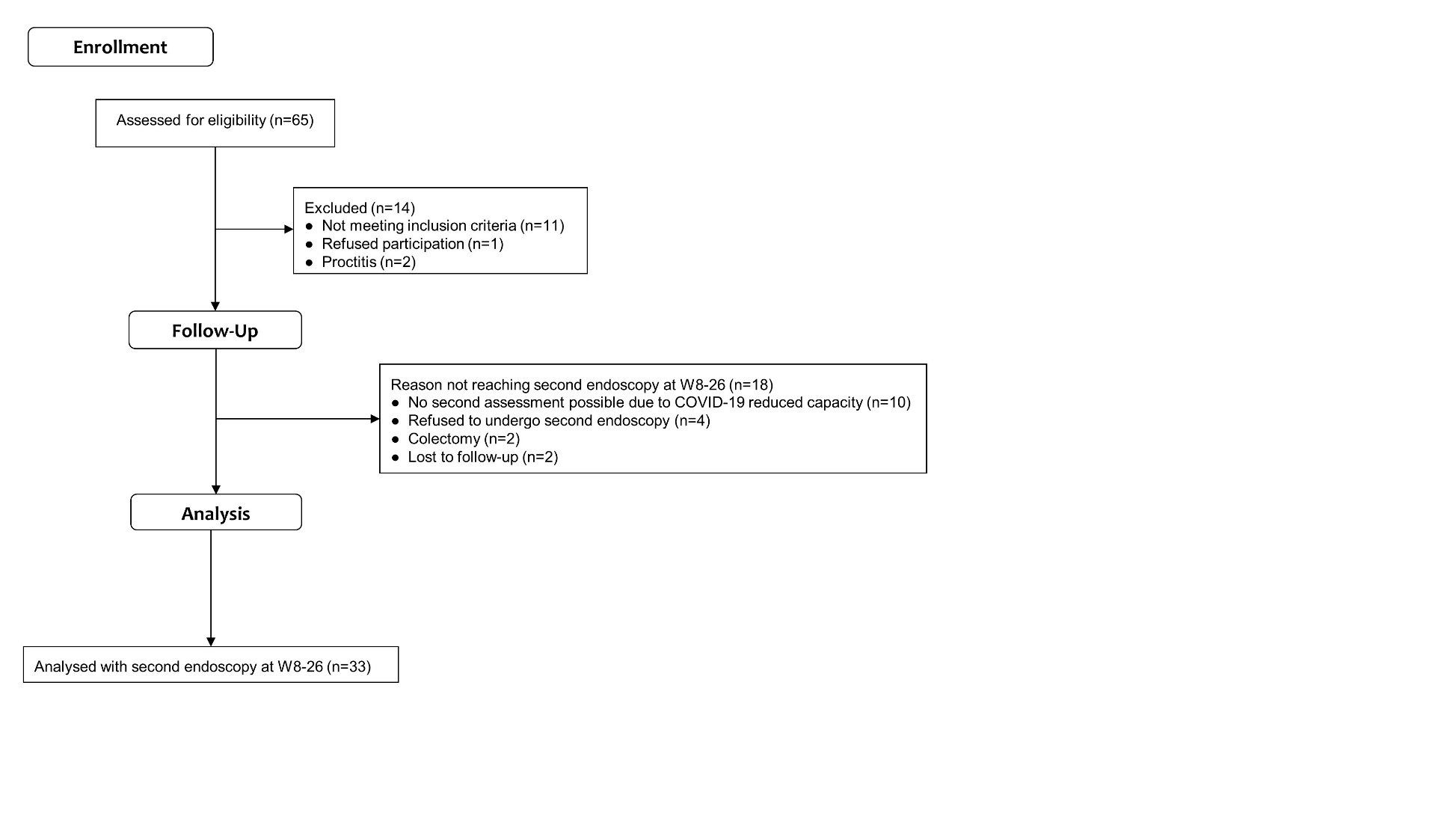


**
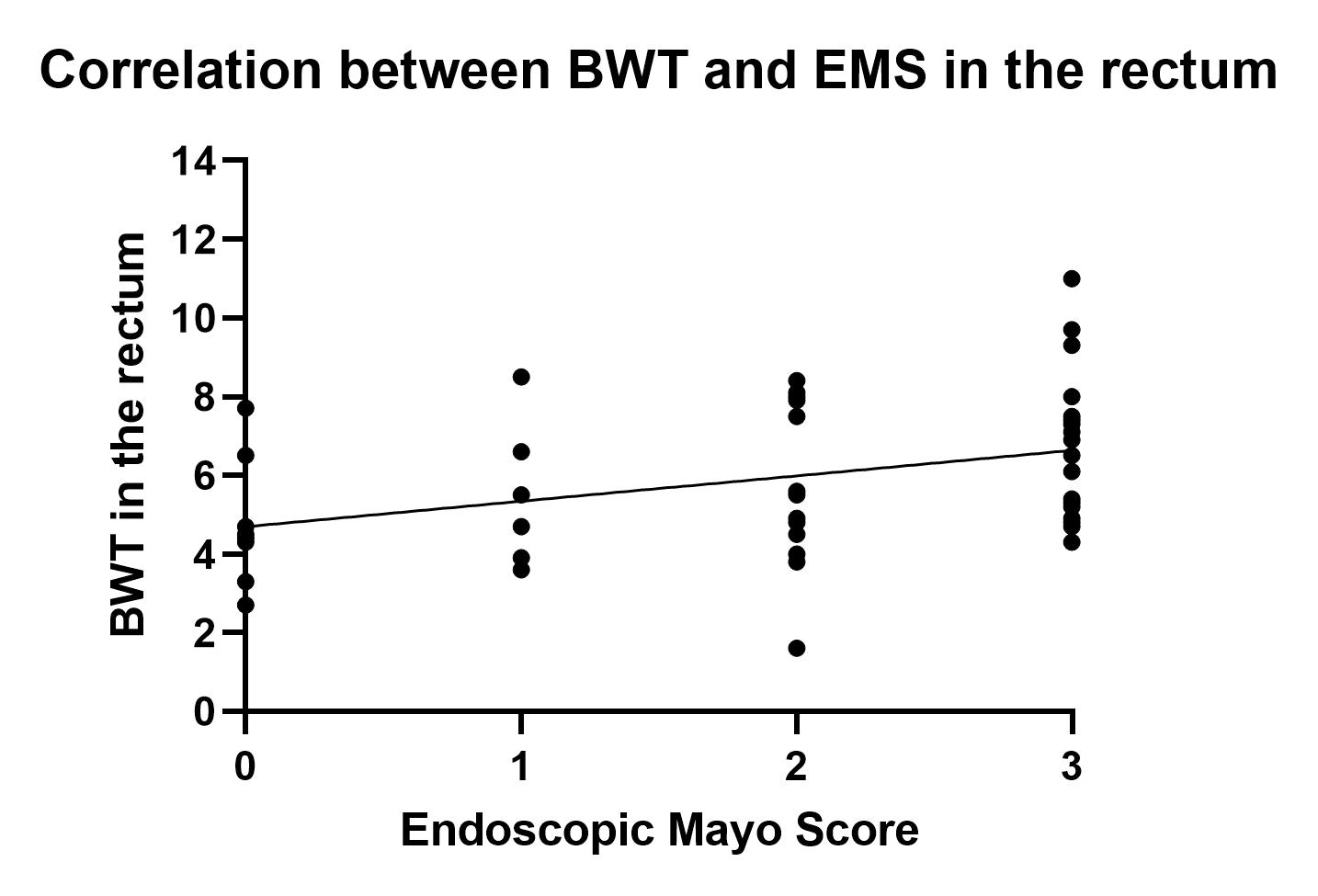
**

A

**
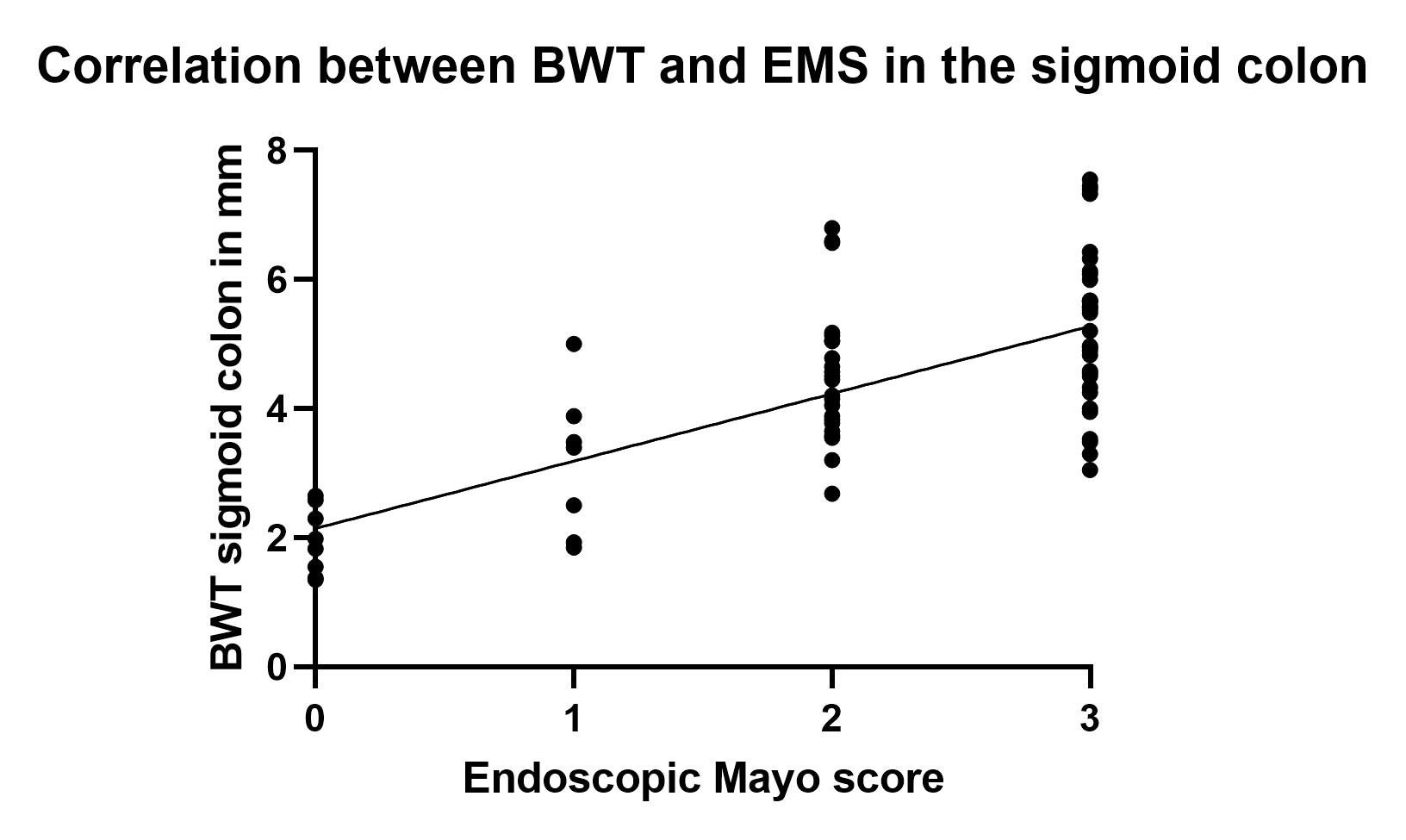
**

**B.**

**C.**


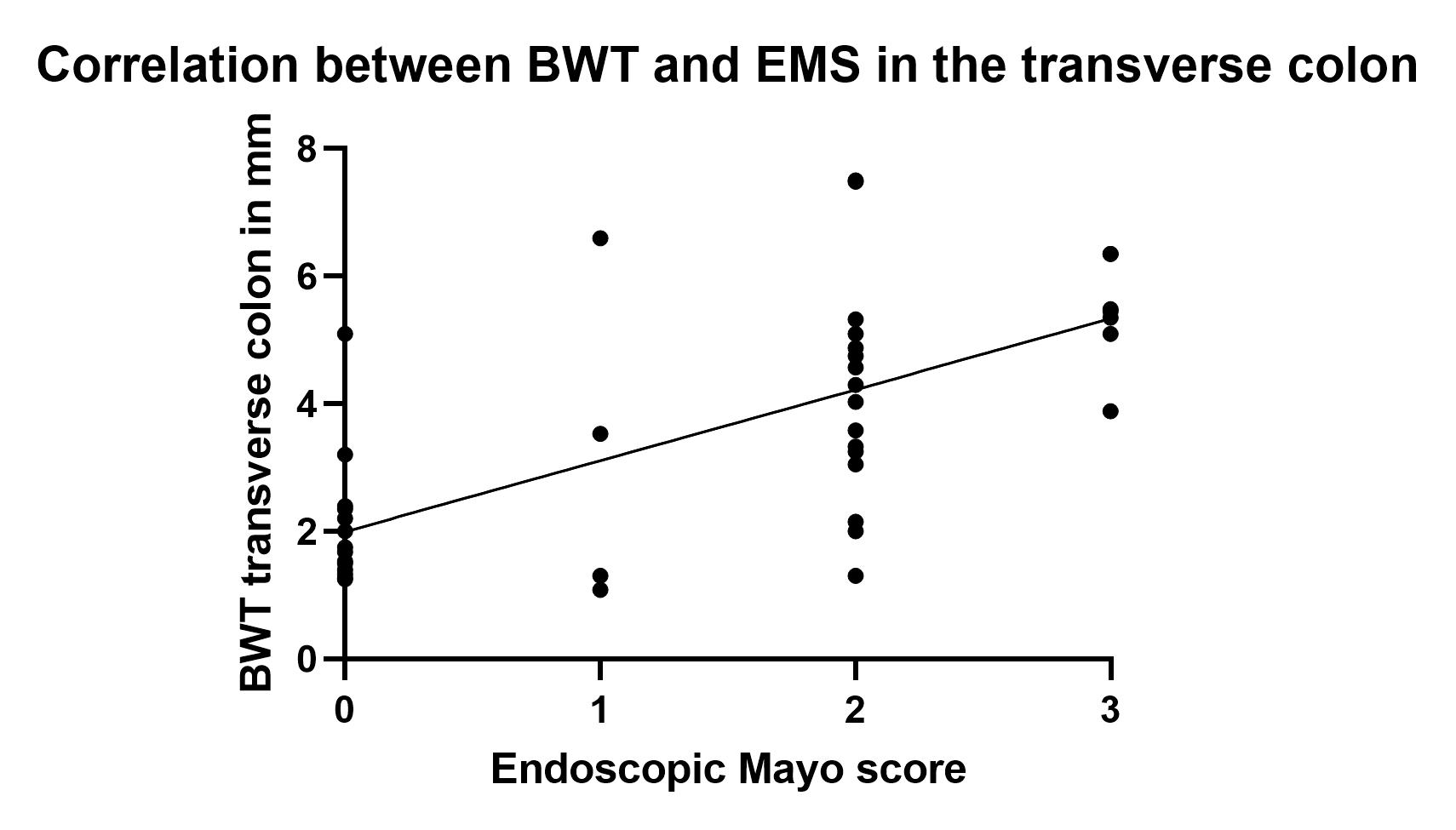


**D.**

**
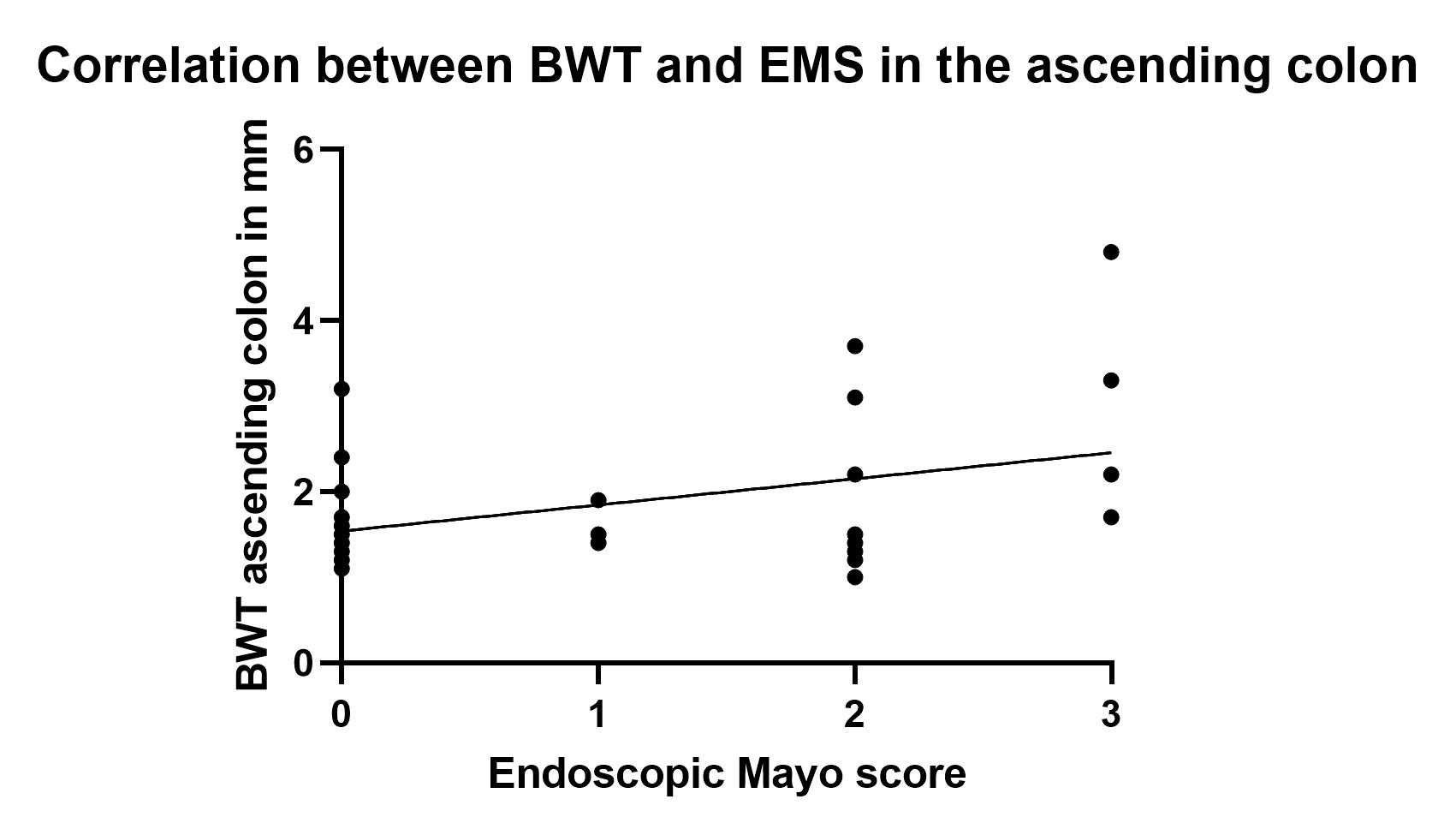
**

**E.**

**Supplementary Figure 3:** Correlation for bowel wall thickness and endoscopic Mayo score in the rectum (ρ=0.38, p=0.008) (A), sigmoid (ρ=0.71, p<0.0001) (B), descending (ρ=0.69, p<0.001 (C), transverse (ρ=0.68, p<0.0001) (D) and ascending (ρ=0.58, p=0.001) (E) colon for both W0 and W8-26. [BWT: bowel wall thickness; EMS: endoscopic Mayo score].


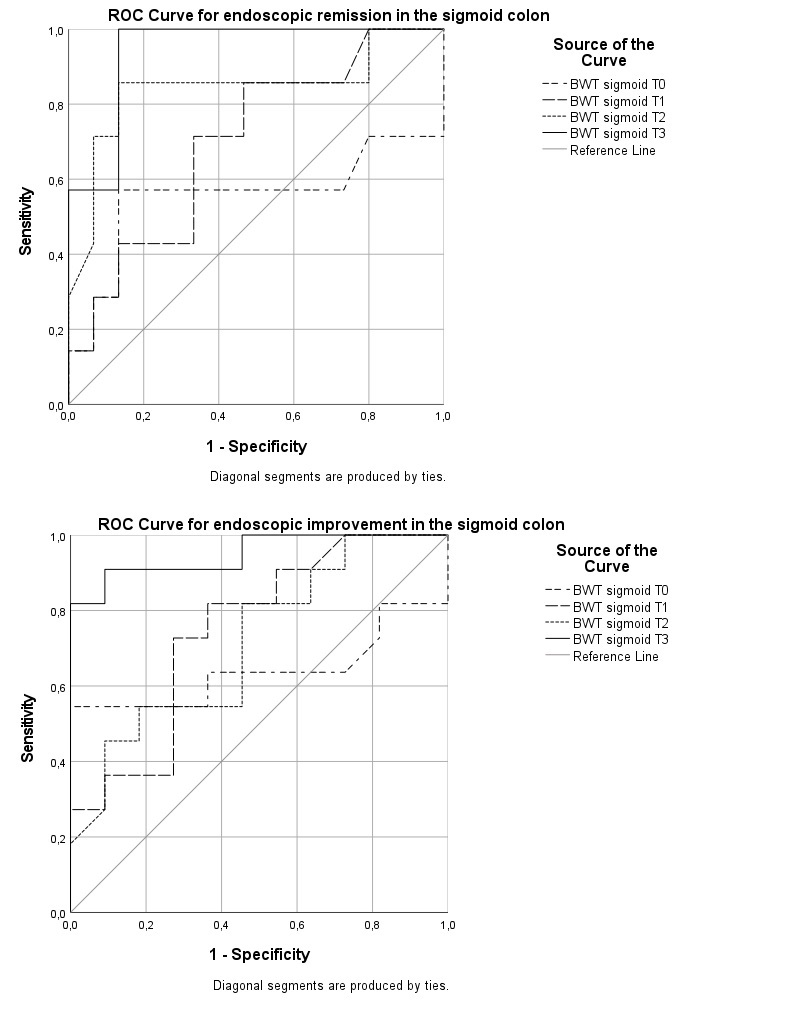


**Supplementary Figure 4**: ROC curves for BWT at several time-points to predict/determine endoscopic remission and endoscopic improvement in the sigmoid colon. [BWT: Bowel wall thickness]. See also Supplementary Table 6.


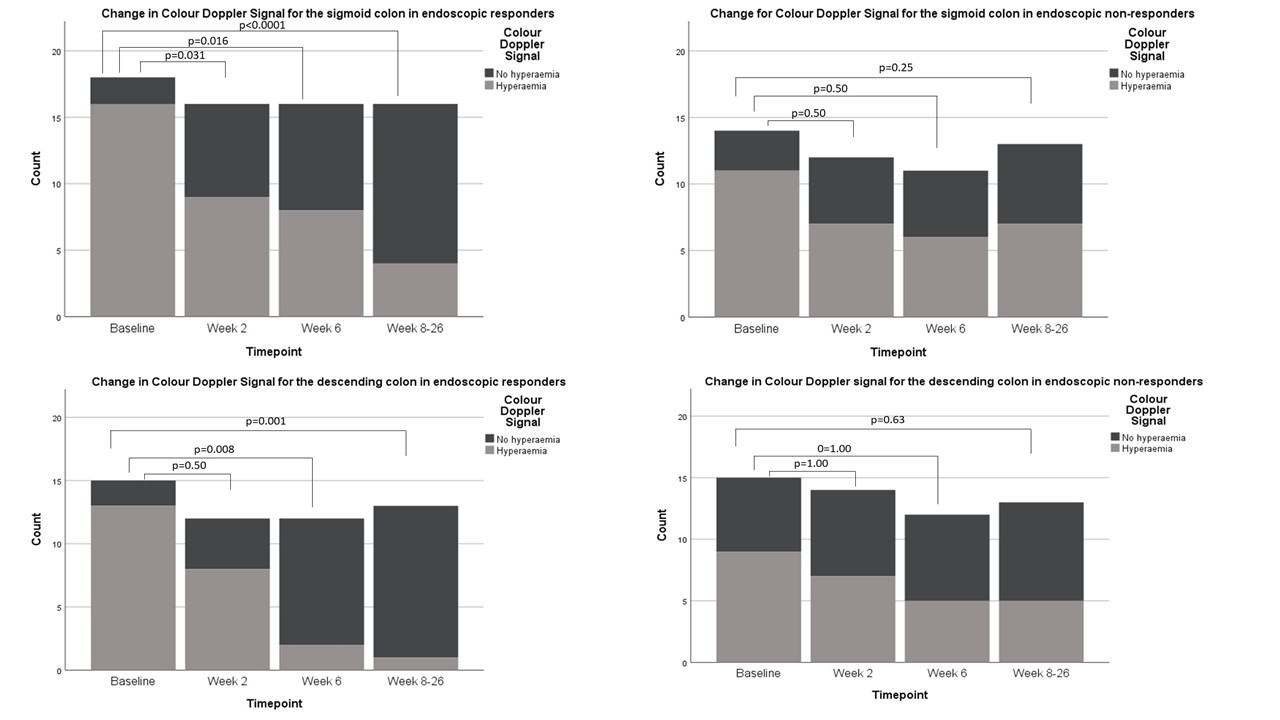


**Supplementary Figure 5**: Paired analysis (McNemar Test) for presence of hyperaemia (CDS≥2) per time-point for sigmoid colon and descending colon in endoscopic responders and non-responders [CDS: Colour Doppler Signal]


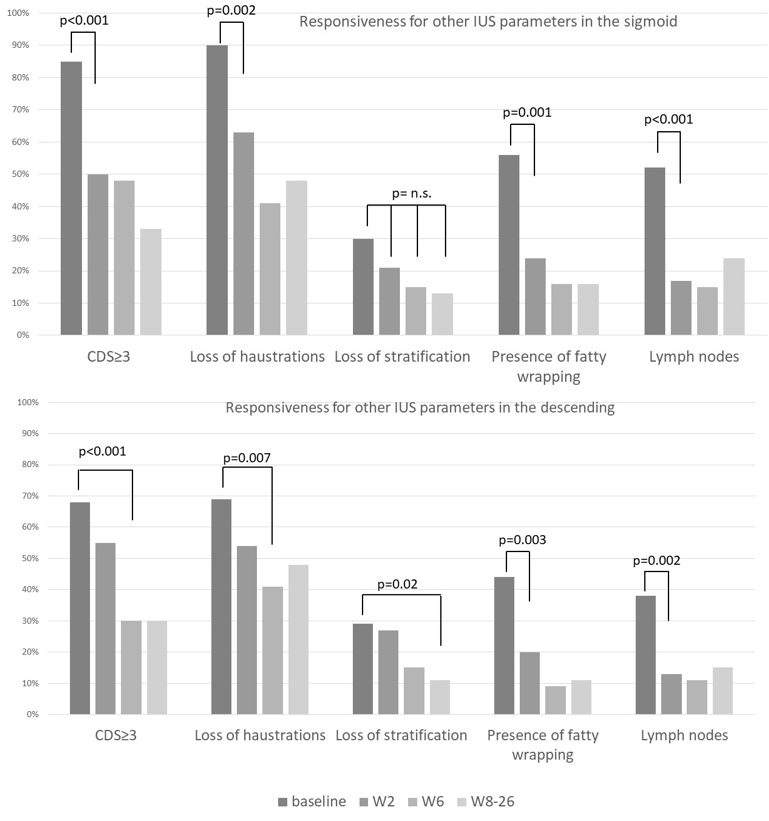


**Supplementary Figure 6:** responsiveness for all other IUS parameters regardless of endoscopic endpoints. In the sigmoid colon all IUS parameters showed most significant decrease at W2. In the descending colon, presence of fatty wrapping and lymph nodes showed most significant decrease at W2, whereas CDS and loss of haustrations were significant as of W6. [CDS: Colour Doppler Signal; IUS: intestinal ultrasound]


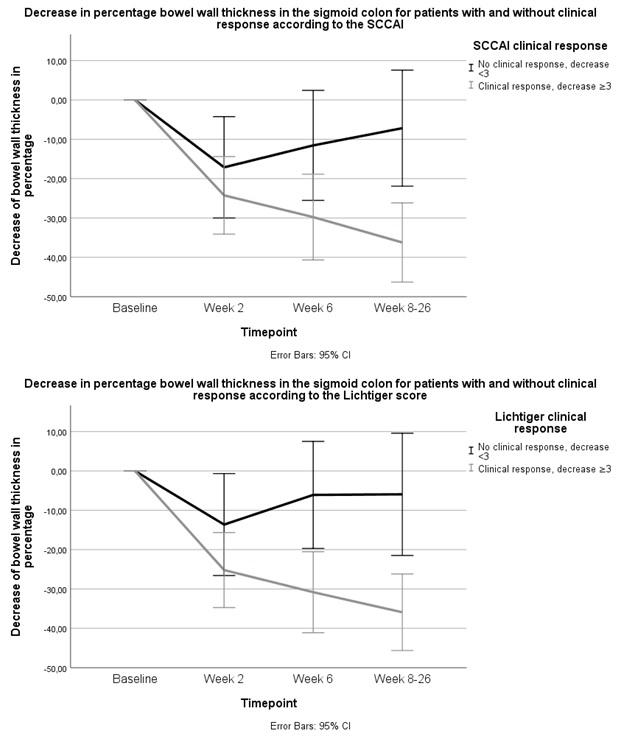


**Supplementary Figure 7:** decrease in mean percentage BWT for patients with and without clinical response according to the SCCAI (W2: -24%±26 vs -17%±26, p=0.37, W6: -30%±30 vs -12%±25, p=0.05 and W8-26: -36%±26 vs -7%±29, p=0.001) and Lichtiger score (W2: -25%±27 vs -14%±23, p=0.16, W6: -31%±29 vs -6%±23, p=0.009 and W8-26: -36%±26 vs -6%±29, p=0.002). [SCCAI: Simple Clinical Colitis Activity Index)
